# Supplementary material for: Evolution of g-type lysozymes in metazoa: insights into immunity and digestive adaptations
Source: Front Cell Dev Biol. 2024 Nov 6;12:1487920. doi: 10.3389/fcell.2024.1487920 (PMC11576321; doi:10.3389/fcell.2024.1487920)
Supplement: Supplementary file 2 [file DataSheet1.PDF]

Supplementary Table 1: The following genomic or transcriptomic assemblies were used for Standalone-BLAST searches:

Taxonomic Group	Species Name	Download Links for Genomic/Transcriptomic Datasets
Holozoans	***Pigoraptor chileana***<br>***Pigoraptor vietnamica***<br>***Ministeria vibrans***	https://doi.org/10.6084/m9.figshare.19895962.v1
Holomycota	***Parvularia atlantis***	https://doi.org/10.6084/m9.figshare.19895962.v1
Ctenophora	***Bolinopsis microptera***	https://www.ncbi.nlm.nih.gov/datasets/genome/?taxon=140493
	Beroe ovata	http://ryanlab.whitney.ufl.edu/bovadb/
	Hormiphora californensis	https://www.ncbi.nlm.nih.gov/datasets/genome/?taxon=140493
	Pleurobrachia bachei	https://www.ncbi.nlm.nih.gov/datasets/genome/?taxon=140493
Aplysia transcriptome dataset	***Aplysia californica***	https://www.ncbi.nlm.nih.gov/geo/query/acc.cgi?acc=GSE79231

Supplementary Table 2: Lysozyme Gene Summary

**Species**	**Lysozyme C**	**Lysozyme G**	**Lysozyme I**
Pomacea canaliculata;Eukaryota; Metazoa; Spiralia; Lophotrochozoa; Mollusca; Gastropoda;Caenogastropoda; Architaenioglossa; Ampullarioidea; Ampullariidae;Pomacea.	Absent	Present	Absent
Saccostrea echinata; Eukaryota; Metazoa; Spiralia; Lophotrochozoa; Mollusca; Bivalvia	Present	Present	Present
Acropora millepora; Eukaryota; Metazoa; Cnidaria; Anthozoa; Hexacorallia; Scleractinia; Astrocoeniina; Acroporidae; Acropora.	Present	Absent	Absent

Actinia tenebrosa; Eukaryota; Metazoa; Cnidaria; Anthozoa; Hexacorallia; Actiniaria; Actiniidae; Actinia.	Present	Absent	Absent
Amphioctopus fangsiao;Eukaryota; Metazoa; Spiralia; Lophotrochozoa; Mollusca;Cephalopoda; Coleoidea; Octopodiformes; Octopoda; Incirrata;_x000B_Octopodidae; Amphioctopus.	Absent	Absent	Present
Aplysia californica	Absent	Present	Absent
Biomphalaria glabrata; Eukaryota; Metazoa; Spiralia; Lophotrochozoa; Mollusca; Gastropoda;Heterobranchia; Euthyneura; Panpulmonata; Hygrophila; Lymnaeoidea;Planorbidae; Biomphalaria.	Absent	Present	Absent
Biomphalaria pfeifferi;Eukaryota; Metazoa; Spiralia; Lophotrochozoa; Mollusca; Gastropoda;Heterobranchia; Euthyneura; Panpulmonata; Hygrophila; Lymnaeoidea;Planorbidae; Biomphalaria.	Absent	Present	Absent
Brachionus calyciflorus; Eukaryota; Metazoa; Spiralia; Gnathifera; Rotifera	Absent	Absent	Present
Brachionus plicatilis; Eukaryota; Metazoa; Spiralia; Gnathifera; Rotifera	Absent	Absent	Present
Branchiostoma belcheri,	Present	Present	Present
Branchiostoma floridae	Present	Present	Present
Branchiostoma lanceolatum	Present	Present	Present
Bulinus truncatus;Eukaryota; Metazoa; Spiralia; Lophotrochozoa; Mollusca; Gastropoda;Heterobranchia; Euthyneura; Panpulmonata; Hygrophila;	Absent	Present	Absent

Lymnaeoidea;Planorbidae; Bulinus.			
Caenorhabditis elegans; Eukaryota; Metazoa; Ecdysozoa; Nematoda;	Absent	Absent	Present
Candidula unifasciata;Eukaryota; Metazoa; Spiralia; Lophotrochozoa; Mollusca; Gastropoda;Heterobranchia; Euthyneura; Panpulmonata; Eupulmonata; Stylommatophora; Helicina; Helicoidea; Geomitridae; Candidula	Absent	Present	Absent
Capitella teleta; Eukaryota; Metazoa; Spiralia; Lophotrochozoa; Annelida	Absent	Absent	Present
Ciona intestinalis;Eukaryota; Metazoa; Chordata; Tunicata; Ascidiacea; Phlebobranchia;Cionidae; Ciona.	Absent	Present	Present
Clavelina lepadiformis; Eukaryota; Metazoa; Chordata; Tunicata; Ascidiacea	Absent	Present	Absent
Conus betulinus;Eukaryota; Metazoa; Spiralia; Lophotrochozoa; Mollusca; Gastropoda;Caenogastropoda; Neogastropoda; Conoidea; Conidae; Conus;Dendroconus.	Absent	Present	Absent
Conus magus;Eukaryota; Metazoa; Spiralia; Lophotrochozoa; Mollusca; Gastropoda;Caenogastropoda; Neogastropoda; Conoidea; Conidae; Conus;Pionoconus.	Absent	Present	Absent
Corticium candelabrum;Eukaryota; Metazoa; Porifera; Homoscleromorpha; Homosclerophorida; Plakinidae; Corticium.	Absent	Present	Absent
Crassostrea angulata; Eukaryota; Metazoa; Spiralia;	Absent	Present	Present

Lophotrochozoa; Mollusca; Bivalvia			
Crassostrea gigas	Absent	Present	Present
Dendronephthya gigantea;Eukaryota; Metazoa; Cnidaria; Anthozoa; Octocorallia;Malacalcyonacea; Nephtheidae; Dendronephthya.	Absent	Present	Absent
Dimorphilus gyrociliatus; Eukaryota; Metazoa; Spiralia; Lophotrochozoa; Annelida; Polychaeta	Absent	Absent	Present
Drosophila melanogaster	Present	Absent	Present
Eisenia andrei: Eukaryota; Metazoa; Spiralia; Lophotrochozoa; Annelida;	Absent	Absent	Present
Elysia chlorotica; Eukaryota; Metazoa; Spiralia; Lophotrochozoa; Mollusca; Gastropoda;Heterobranchia; Euthyneura; Panpulmonata; Sacoglossa; Placobranchoidea; Plakobranchidae; Elysia.	Absent	Present	Absent
Elysia crispata; Eukaryota; Metazoa; Spiralia; Lophotrochozoa; Mollusca; Gastropoda;Heterobranchia; Euthyneura; Panpulmonata; Sacoglossa; Placobranchoidea; Plakobranchidae; Elysia.	Absent	Present	Absent
Gigantopelta aegis;Eukaryota; Metazoa; Spiralia; Lophotrochozoa; Mollusca; Gastropoda	Absent	Absent	Present
Haliotis discus discus; Eukaryota; Metazoa; Spiralia; Lophotrochozoa; Mollusca; Gastropoda;	Present	Present	Present
Haliotis discus hannai; Eukaryota; Metazoa; Spiralia; Lophotrochozoa; Mollusca; Gastropoda;	Present	Absent	Absent
Haliotis diversicolor; Eukaryota; Metazoa; Spiralia;	Present	Present	Present

Lophotrochozoa; Mollusca; Gastropoda			
Haliotis rufescens; Eukaryota; Metazoa; Spiralia; Lophotrochozoa; Mollusca; Gastropoda;	Present	Present	Present
Homo sapiens	Present	Present	Absent
Lingula anatina; Eukaryota; Metazoa; Spiralia; Lophotrochozoa; Brachiopoda;	Absent	Present	Absent
Littorina saxatilis;Eukaryota; Metazoa; Spiralia; Lophotrochozoa; Mollusca; Gastropoda;Caenogastropoda; Littorinimorpha; Littorinoidea; Littorinidae;_x000B_Littorina.	Absent	Present	Absent
Lottia gigantea	Present	Present	Absent
Mercenaria mercenaria	Present	Absent	Present
Meretrix meretrix (Asiatic hard clam)	Absent	Absent	Present
Mizuhopecten yessoensis;Eukaryota; Metazoa; Spiralia; Lophotrochozoa; Mollusca; Bivalvia;Autobranchia; Pteriomorphia; Pectinida; Pectinoidea; Pectinidae;Mizuhopecten.	Absent	Present	Present
Mya arenaria	Absent	Absent	Present
Mytilus galloprovincialis; Eukaryota; Metazoa; Spiralia; Lophotrochozoa; Mollusca; Bivalvia	Present	Present	Present
Octopus bimaculoides; Eukaryota; Metazoa; Spiralia; Lophotrochozoa; Mollusca; Cephalopoda	Absent	Absent	Present
Octopus sinensis;Eukaryota; Metazoa; Spiralia; Lophotrochozoa; Mollusca;Cephalopoda; Coleoidea; Octopodiformes; Octopoda; Incirrata;_x000B_Octopodidae; Octopus.	Absent	Absent	Present

Octopus vulgaris;Eukaryota; Metazoa; Spiralia; Lophotrochozoa; Mollusca;Cephalopoda; Coleoidea; Octopodiformes; Octopoda; Incirrata; Octopodidae; Octopus.	Absent	Absent	Present
Ostrea edulis; Eukaryota; Metazoa; Spiralia; Lophotrochozoa; Mollusca; Bivalvia	Absent	Present	Present
Owenia fusiformis; Eukaryota; Metazoa; Spiralia; Lophotrochozoa; Annelida; Polychaeta;	Present	Absent	Absent
Patella caerulea; Eukaryota; Metazoa; Spiralia; Lophotrochozoa; Mollusca; Gastropoda	Present	Present	Absent
Patella vulgata; Eukaryota; Metazoa; Spiralia; Lophotrochozoa; Mollusca; Gastropoda;	Present	Present	Absent
Patelloida mimula;Eukaryota; Metazoa; Spiralia; Lophotrochozoa; Mollusca; Gastropoda;Patellogastropoda; Lottioidea; Lottiidae; Patelloida.	Absent	Present	Absent
Pecten maximus;_x000B_Eukaryota; Metazoa; Spiralia; Lophotrochozoa; Mollusca; Bivalvia;_x000B_Autobranchia; Pteriomorphia; Pectinida; Pectinoidea; Pectinidae; Pecten.	Absent	Present	Present
Penaeus chinensis; Eukaryota; Metazoa; Ecdysozoa; Arthropoda; Crustacea;	Present	Absent	Present
Penaeus indicus; Eukaryota; Metazoa; Ecdysozoa; Arthropoda; Crustacea	Present	Absent	Present
Penaeus japonicus; Eukaryota; Metazoa; Ecdysozoa; Arthropoda; Crustacea;	Present	Absent	Present

Penaeus monodon; Eukaryota; Metazoa; Ecdysozoa; Arthropoda; Crustacea;	Present	Absent	Present
Penaeus vannamei; Eukaryota; Metazoa; Ecdysozoa; Arthropoda; Crustacea;	Present	Absent	Present
Physella acuta; Eukaryota; Metazoa; Spiralia; Lophotrochozoa; Mollusca; Gastropoda; Heterobranchia; Euthyneura; Panpulmonata; Hygrophila; Lymnaeoidea; Physidae; Physella.	Absent	Present	Absent
Porites evermanni; Eukaryota; Metazoa; Cnidaria; Anthozoa; Hexacorallia; Scleractinia; Fungiina; Poritidae; Porites	Present	Absent	Absent
Porites lobata; Eukaryota; Metazoa; Cnidaria; Anthozoa; Hexacorallia; Scleractinia; Fungiina; Poritidae; Porites	Present	Absent	Absent
Rotaria sp. Silwood1; Eukaryota; Metazoa; Spiralia; Gnathifera; Rotifera; Eurotatoria; Bdelloidea; Rotaria	Absent	Absent	Present
Saccoglossus kowalevskii; Eukaryota; Metazoa; Hemichordata	Absent	Absent	Present
Saccostrea cucullata; Eukaryota; Metazoa; Spiralia; Lophotrochozoa; Mollusca; Bivalvia	Present	Present	Present
Saccostrea echinata; Eukaryota; Metazoa; Spiralia; Lophotrochozoa; Mollusca; Bivalvia	Present	Present	Present
Strongylocentrotus purpuratus	Absent	Absent	Present
Styela clava; Eukaryota; Metazoa; Chordata; Tunicata; Ascidiacea;	Absent	Present	Absent
Suberites domuncula; Eukaryota; Metazoa; Porifera; Demospongiae	Absent	Absent	Present
Trichoplax adhaerens; Eukaryota; Metazoa; Placozoa;	Absent	Present	Absent

Uniplacotomia; Trichoplacea; Trichoplacidae; Trichoplax			
Trichoplax sp. H2 MB-2014; Eukaryota; Metazoa; Placozoa; Uniplacotomia; Trichoplacea; Trichoplacidae; Trichoplax	Absent	Present	Absent
Xenia sp.	Absent	Present	Present

Newick Tree:

(Cc_94995:0.69467766,(Cc_98523:0.14062073,Cc_98591:0.20856957)0.977500:0.68948398,(((
((Myx_gewl:0.32068740,Myx_34660:0.32995952)0.790600:0.56860326,((Hya_gewl:0.0631147
9,Sa_gewl:0.10662738)0.993200:0.35038701,(Pca_Gewl:0.02614895,(Pyx_gewl:0.00000001,Ar
c_gewl:0.00000001)0.825000:0.01258754)0.937000:0.17525471)0.956400:0.62920286)0.99960
0:1.11080403,(((((Hdd_lyzg:0.04676961,(Hdi_LysG1:0.05741942,Hr_78614:0.04310384)0.414
400:0.04646386)0.999500:0.25389068,((Pc_09293:0.25179882,Pv_09293:0.03172316)0.862300
:0.05637611,(Pm_gewl:0.15612213,Lg_LYZg1:0.11817197)0.936300:0.06685349)0.984800:0.1
3217851)0.786400:0.06699824,(Ac_LYZg3:0.97046241,(Ls_01764:0.08853191,(Cm_LYZg2:0.
12884973,Cm_LYZT3a:0.11387634)1.000000:0.77377059)0.994200:0.29312016)0.778300:0.1
3597599)0.959500:0.36700152,(((My_59246:0.34430781,Pm_29535:0.19159531)0.999000:0.58
964234,((Bg_LYZg3:0.01846305,Bp_08986:0.03507887)1.000000:0.74278169,((((Bg_LYZg2:
0.04589353,Bp_08972:0.01737844)0.999500:0.65077230,(Bt_15341:0.59776409,((Bt_31685:0.
02958545,Bt_43005:0.03393599)0.999500:0.46580330,(Bg_LYZg1:0.04771386,(Bp_05359:0.0
6417651,Bf_05346:0.04752128)0.697300:0.01231964)0.981400:0.28206647)0.954600:0.21827
226)0.243900:0.08113125)0.998200:0.58306219,((Pc_57781:0.89490155,(Pc_57861:0.3958976
7,Ls_08512:0.31794406)0.385200:0.29035012)0.998300:0.65272802,((Ac_LYZg2:0.28110074,
Ec_42267:0.96102748)0.625400:0.15181148,(Ac_LYZg1:0.99039959,(Ech_17175:0.45459169,
Ec_47861:0.48153219)0.999600:0.78983718)0.837700:0.15233911)0.884600:0.15088219)0.581
100:0.09693833)0.885800:0.12262261,(Pa_37057:0.25860833,(Pa_34965:0.15060654,((Bp_089
85:0.01789046,(Bg_LYZg6:0.00000001,Bg_LYZg7:0.00000001)0.000000:0.00296587)0.99980
0:0.44537281,(Pa_44383:0.17187248,(Cu_13863:0.89695282,(Bg_LYZg5:0.15087097,Bg_LYZ
g4:0.12209748)0.704100:0.11162359)0.271600:0.08160503)0.947900:0.17664087)0.996500:0.3
2063334)0.456500:0.07389877)0.693000:0.13592159)0.479600:0.05667264)0.985200:0.324904
01)0.909600:0.23963086,((Mg_gewl:0.00944019,Mg_gewl2:0.00000001)1.000000:0.80731674,
(Se_85121:0.83872517,(Oe_46487:0.92735509,(Se_82739:0.83918527,(Cg_26612:0.00000001,
Ca_78719:0.00000001)0.999700:1.21815504)0.894200:0.40664154)0.996500:0.82023705)0.52
8300:0.15498560)0.584700:0.11040593)0.920600:0.25060543)0.826800:0.22571942,(Lg_LYZg
2:0.99983055,Lg_LYZg3:0.43779359)0.885500:0.33755532)0.999800:1.07839816)0.364600:0.
06457111,(Tsp_Lys:0.00000001,Ta_63166:0.01339605)0.999700:0.91679005)0.824900:0.1710
5538,(((Bf_LYZg4:0.28164934,(Bf_LYZg2:0.32054650,(Bf_LYZg5:0.47195028,Bf_LYZg1:0.
33275005)0.817800:0.13167609)0.940000:0.23247909)0.975300:0.40466965,(La_79286:0.7905
8002,Bf_LYZg3:0.65954433)0.999400:1.03284245)0.018000:0.09261903,((Cl_10499:0.895717
18,(Ci_76087:0.94434451,(Sc_LYZT3a:0.40722713,Cl_S3624:0.31118062)0.853000:0.169342

19)0.972000:0.32458949)0.034600:0.30549762,(Bf_LYZg6:0.90224634,((Xsp_46186:0.40612300,Dg_27517:0.76112546)0.996800:1.12612853,(Hs_LYG1:1.90706386,Hs_LYG2:1.26211524)0.966400:0.91868460)0.832000:0.29955962)0.622500:0.25060351)0.815300:0.35776075)0.914500:0.39087745)1.000000:2.82852883);

Stats for the Newick Tree:

```
oooooooooooooooooooooooooooooooooooooooooooooooooooooooooooooooooooooooooooooooooooooooooooooooooo
                                 ---  PhyML 3.3.20220408  ---
                             http://www.atgc-montpellier.fr/phyml
                          Copyright CNRS - Universite Montpellier
oooooooooooooooooooooooooooooooooooooooooooooooooooooooooooooooooooooooooooooooooooooooooooooooooo

. Sequence filename:                    lysozyme_gewl_muscle_phy
. Data set:                             #1
. Initial tree:                         BioNJ
. Model of amino acids substitution:    WAG
. Number of taxa:                       76
. Log-likelihood:                       -25712.04529
. Unconstrained log-likelihood:         -6514.08342
. Composite log-likelihood:             -219126.48510
. Parsimony:                            5061
. Tree size:                            55.79731
. FreeRate model:                       Yes
  - Number of classes:                  4
  - Relative rate in class 1:           1.59912 [freq=0.492083]
  - Relative rate in class 2:           0.59037 [freq=0.306499]
  - Relative rate in class 3:           0.20798 [freq=0.143962]
  - Relative rate in class 4:           0.03850 [freq=0.057456]

. Run ID:                               none
. Random seed:                          1712866370
. Subtree patterns aliasing:            no
. Version:                              3.3.20220408
. Time used:                            0h10m43s (643 seconds)
```

oooooooooooooooooooooooooooooooooooooooooooooooooooooooooooooooooooooo
oooooooooooooooooooooo
Suggested citations:
S. Guindon, JF. Dufayard, V. Lefort, M. Anisimova, W. Hordijk, O. Gascuel
"New algorithms and methods to estimate maximum-likelihood phylogenies: assessing the performance of PhyML 3.0."
Systematic Biology. 2010. 59(3):307-321.

S. Guindon & O. Gascuel
"A simple, fast, and accurate algorithm to estimate large phylogenies by maximum likelihood"
Systematic Biology. 2003. 52(5):696-704.

Ooooooooooooooooooooooooooooooooooooooooooooooooooooooooooooooooooooooo
oooooooooooooooooooooo

Alignment file:

```
CLUSTAL 2.1 multiple sequence alignment


Cc_94995        ------------------------------------------------------------
Cc_98523        ------------------------------------------------------------
Cc_98591        ------------------------------------------------------------
Hs_LYG1         ------------------------------------------------------------
Hs_LYG2         ------------------------------------------------------------
Dg_27517        ------------------------------------------------------------
Xsp_46186       ------------------------------------------------------------
Myx_gewl        ------------------------------------------------------------
Myx_34660       ------------------------------------------------------------
Bf_LYZg6        ------------------------------------------------------------
Bf_LYZg3        ------------------------------------------------------------
La_79286        ------------------------------------------------------------
Bf_LYZg1        ------------------------------------------------------------
Bf_LYZg5        ------------------------------------------------------------
Bf_LYZg4        ------------------------------------------------------------
Bf_LYZg2        ------------------------------------------------------------
Ci_76087        ------------------------------------------------------------
Sc_LYZT3a       ---------------------------------------------MKIVVVLALIGLAAA
Cl_S3624        ------------------------------------------MKVAFFGFLLSVVNSARS
Cl_10499        ----------------------------------------MEFHLVYVMVFCAAIFGVGN
Tsp_Lys         ------------------------------------------------------------
Ta_63166        ------------------------------------------------------------
Pca_Gewl        ------------------------------------------------------------
Pyx_gewl        ------------------------------------------------------------
Arc_gewl        ------------------------------------------------------------
Sa_gewl         ------------------------------------------------------------
Hya_gewl        ------------------------------------------------------------
Cg_26612        ------------------------------------------------------------
Ca_78719        ------------------------------------------------------------
Se_82739        ------------------------------------------------------------
Oe_46487        ------------------------------------------------------------
Pc_57781        ------------------------------------------------------------
Pc_57861        ------------------------------------------------------------
Ls_08512        ------------------------------------------------------------
Ec_47861        ------------------------------------------------------------
Ech_17175       ------------------------------------------------------------
Bg_LYZg2        ------------------------------------------------------------
```

```
Bp_08972        ------------------------------------------------------------
Bt_15341        MYLTDVPHRSTSQMYLTEIPHRSTSQKYLTVVPHRSTSQKYLTDVPHRNTSQKYFTKVPH
Bt_43005        ------------------------------------------------------------
Bt_31685        ------------------------------------------------------------
Bg_LYZg1        ------------------------------------------------------------
Bp_05359        ------------------------------------------------------------
Bf_05346        ------------------------------------------------------------
Bg_LYZg3        ------------------------------------------------------------
Bp_08986        ------------------------------------------------------------
Ac_LYZg1        ------------------------------------------------------------
Ec_42267        ------------------------------------------------------------
Cu_13863        ------------------------------------------------------------
Bg_LYZg6        ------------------------------------------------------------
Bg_LYZg7        ------------------------------------------------------------
Bp_08985        ------------------------------------------------------------
Ac_LYZg2        ------------------------------------------------------------
Pa_37057        ------------------------------------------------------------
Pa_34965        ------------------------------------------------------------
Pa_44383        ------------------------------------------------------------
Bg_LYZg4        ------------------------------------------------------------
Bg_LYZg5        ------------------------------------------------------------
Se_85121        ------------------------------------------------------------
Pm_29535        ------------------------------------------------------------
My_59246        ------------------------------------------------------------
Mg_gewl         ------------------------------------------------------------
Mg_gewl2        ------------------------------------------------------------
Lg_LYZg2        ------------------------------------------------------------
Ls_01764        ------------------------------------------------------------
Cm_LYZT3a       -------------------------------------------MKWVGFLVWCGLLFCVR
Cm_LYZg2        ------------------------------------------------------------
Ac_LYZg3        ------------------------------------------------------------
Lg_LYZg3        ------------------------------------------------------------
Hdd_lyzg        ------------------------------------------------------------
Hr_78614        ------------------------------------------------------------
Hdi_LysG1       ------------------------------------------------------------
Pm_gewl         ------------------------------------------------------------
Lg_LYZg1        ------------------------------------------------------------
Pc_09293        ------------------------------------------------------------
Pv_09293        ------------------------------------------------------------


Cc_94995        ---------------------------------------------------MHREHSISF
Cc_98523        ------------------------------------------------------------
Cc_98591        ------------------------------------------------------------
Hs_LYG1         ------------------------------------------------------------
Hs_LYG2         ------------------------------------------------------------
Dg_27517        ------------------------------------------------------------
Xsp_46186       ------------------------------------------------------------
Myx_gewl        ------------------------------------------------------------
Myx_34660       ------------------------------------------------------------
Bf_LYZg6        ------------------------------------------------------------
Bf_LYZg3        ----------MKALCVFVVGLLLVTEALCAVSRSDFARIMCLKRRSLDGVLGRSKRCAGG
La_79286        --------MLLKRCSFILLALSLYTADVLGRTSNHARILCTRKRRDLQGLSFHERLKRCS
Bf_LYZg1        ------------------------------------------------------------
Bf_LYZg5        ----------------MLFLVILAVAGVATADDWQCTTSLGGNQQFGQCAHVDSCPYSYY
Bf_LYZg4        ------------------------------------------------------------
Bf_LYZg2        ------------------------------------------------------------
Ci_76087        ------------------------------------------------------------
Sc_LYZT3a       SDWGCTKDGGTCQDYRNAVCTAGYETGLCDGDSNRRCCLQCSASCASSEAMYSQN-DGEC
Cl_S3624        SDLSCTLQGGTCLDYRYYFCTAGFEQGLCDGDSNRKCCLECDQTCLSEENQYAQCCDSEC
Cl_10499        TDTSCKKLGGKCVDWRYNICTNGVRRNLCRGDSNRRCCFRCASTPCVIRENRWKASDGPC
Tsp_Lys         ------------MKSVILLSAFVAVAFAALNDDCNAGQGSCQYDSQCYTGTPASGLCPYD
Ta_63166        ------------------------------------------------------------
Pca_Gewl        ------------------------------------------------------------
Pyx_gewl        ------------------------------------------------------------
Arc_gewl        ------------------------------------------------------------
Sa_gewl         -----------------------------------------------------------M
Hya_gewl        --------------------------------------------------------MTTT
Cg_26612        ------------------------------------------------------------
Ca_78719        ------------------------------------------------------------
Se_82739        ------------------------------------------------------------
```

```
Oe_46487        -----------MPAIASLSYVFLLSVLAQNILGYELMNLVKEFYIKDQVSCRSVGGKCQN
Pc_57781        ------------------------------------------------------------
Pc_57861        ---------------------------------------------MYKGPANRTTSIFLD
Ls_08512        ------------------------------------------------------------
Ec_47861        ----------------------------------------------------------ME
Ech_17175       ---------------------------------------------MHTGLITLITFSCSV
Bg_LYZg2        ------------------------------------------------------------
Bp_08972        ------------------------------------------------------------
Bt_15341        RCTSQKYLTEVPYRSRPTLQKYLTEVLHKSTSQSHQRRPCCKYSGVGLSHILLQFKLKQL
Bt_43005        ------------------------------------------------------------
Bt_31685        ------------------------------------------------------------
Bg_LYZg1        ------------------------------------------------------------
Bp_05359        ------------------------------------------------------------
Bf_05346        ------------------------------------------------------------
Bg_LYZg3        ------------------------------------------------------------
Bp_08986        ------------------------------------------------------------
Ac_LYZg1        ---------------MITLLLLVSLAVTTNTDAFFFRSWTRSPPVRSPITRSPFWWTWAP
Ec_42267        -------------------------MRCCQQYKIKRITEHAPLRIHLDLIRCSHIPGNSQ
Cu_13863        ------------------------------------------------------------
Bg_LYZg6        ------------------------------------------------------------
Bg_LYZg7        ------------------------------------------------------------
Bp_08985        ------------------------------------------------------------
Ac_LYZg2        ------------------------------------------------------------
Pa_37057        ------------------------------------------------------------
Pa_34965        ------------------------------------------------------------
Pa_44383        ------------------------------------------------------------
Bg_LYZg4        ------------------------------------------------------------
Bg_LYZg5        ------------------------------------------------------------
Se_85121        ------------------------------------------------------------
Pm_29535        -----------------------------------------------MPLACLQILHVTF
My_59246        --------------------------------------------------MALTSLQDIR
Mg_gewl         ------------------------------------------------------------
Mg_gewl2        ------------------------------------------------------------
Lg_LYZg2        ---------------MSKMGYMAYILVVFALLCFYVNADQCTYRGGQCKRSSGGWWFWSR
Ls_01764        ----------------MLALILLSVLSVALGDQCTSHVLTSGSHSGAHGIGCVKSGCCQF
Cm_LYZT3a       GSDVPCRQAGGTCQHNSLYCSGSYRRNMCAGSARRQCCIPSRASSGSDTPCINQGGTCQY
Cm_LYZg2        ----------------------------------------RRASSGSDTPCINKGGTCQY
Ac_LYZg3        ------------------------------------------------------------
Lg_LYZg3        ------------------------------------------------------------
Hdd_lyzg        ---------------MHIFLVLTAAVAAVNGDACTSHVLT-GTHAGTHGVGCMKASCCAD
Hr_78614        ---------------MRTFLLLIVAVAAVNGDACTSHVLT-GTHAGSQGVGCMKASCCAN
Hdi_LysG1       ---------------MRTFLLLTIAVAAVNGDACTSHVLT-GTHAGTQGVGCMEASCCAN
Pm_gewl         ------------------------IISAAAGDQCTSHALTSGSHKGTHGIGCVKSGCCAN
Lg_LYZg1        ------------------------------------------------------------
Pc_09293        ------------------------------------------------------------
Pv_09293        ----------------------------VQGDSCTSHVLT-GSHAGAKGIGCVKSGCCAN


Cc_94995        IIIIIMLQLAVVLINLLLF-----------------------------------------
Cc_98523        --MVPSSVVLINLFLFVYASNGYVMVENSQPPSL--------------------------
Cc_98591        --MVPSSFVLINLFLFVYASNGFVM-----------------------------------
Hs_LYG1         ------MSALWLLLGLLALM----------------------------------------
Hs_LYG2         ---MLSSVVFWGLIALIGTSRG--------------------------------------
Dg_27517        ------------------------------------------------------------
Xsp_46186       ------------------------------------------------------------
Myx_gewl        -----MSNNLSVRTSSLARTQT--------------------------------------
Myx_34660       -----MSATTSQRTNSVASTRSQ-------------------------------------
Bf_LYZg6        ------------------------------------------------------------
Bf_LYZg3        SGGGGGGISIDETITVIGQRIHDPFVHVISDPGAIRDFLGSFGGNGGGLDH---------
La_79286        GGGGGGIETITVVGQRISDPFVTVIRGDAIRGFLDSFAGQG------VITH---------
Bf_LYZg1        -------MLFVVLSAFV-------------------------------------------
Bf_LYZg5        VSNKCPSYGNDIKCCYNCHLGGCSTSGGGST-----------------------------
Bf_LYZg4        ------------------------------------------------------------
Bf_LYZg2        -----MGMNHYIARTFL-------------------------------------------
Ci_76087        ------MLKVFLLFLFV-------------------------------------------
Sc_LYZT3a       DTAGGDCHHNSNYCDGTYYNGKCGGPSERQCCVSSGG-----------------------
Cl_S3624        TNDGGKCQLNSNYCSGSYATGKCGGPYNRQCCSTS-------------------------
Cl_10499        TNREGKCQQNSNFCRVSYHSGLCGGPTTRQCCVETSTPSGASDSQCTVRGGQCQLDSNHC
Tsp_Lys         PSNVKCCPKVGQSCKSSTGNCMLTTHCGGTTYS---------------------------
Ta_63166        ------------------------------------------------------------
Pca_Gewl        -----MTTTSAVRASQTQATRI--------------------------------------
```

```
Pyx_gewl        -----MTTTSAVRASQTQATR---------------------------------------
Arc_gewl        -----MTTTSAVRASQTQATR---------------------------------------
Sa_gewl         TTASATRSQNTNATRAT-------------------------------------------
Hya_gewl        SSTRSQSSQSTQSTRST-------------------------------------------
Cg_26612        ---MVTWLWVFTIITII-------------------------------------------
Ca_78719        ---MVTWLWVFTIITII-------------------------------------------
Se_82739        ----MKTFILLATCILVALN----------------------------------------
Oe_46487        ATGQCHGSYISSNCDGVAGLKCCSPKTQVQDTGDCPGLPILSRDTWGAAIPKAIEKLALP
Pc_57781        -----MLLTLSVLVVTL-------------------------------------------
Pc_57861        FPAITNFTMLLTLSVFVLT-----------------------------------------
Ls_08512        ------MYLLLCLVASL-------------------------------------------
Ec_47861        VTMLLASCFAFVFTSVMAVEMTIIETREVFSKLDLQSVRRVNS-----------------
Ech_17175       LAITDVSALELEVEMVITETRRVYPRPDSRQPGRHANTT---------------------
Bg_LYZg2        -------MRLLILTTLVVATYAGLADKKEKK-----------------------------
Bp_08972        -------MRLLILTTLVVATYAGLADKIEKK-----------------------------
Bt_15341        NGQDKTWPFLSLATAVVRILEWVCPTHILLQFKLKQSNGHDKTWPFPQNKMQVVWILLSF
Bt_43005        ------MMNVILLLPLV-------------------------------------------
Bt_31685        ------MVNVILLLPLV-------------------------------------------
Bg_LYZg1        -------MFLLLLFSVL-------------------------------------------
Bp_05359        -------MFLLLLFSVL-------------------------------------------
Bf_05346        -------MFLLLLFSVL-------------------------------------------
Bg_LYZg3        -------MFVIVLCALV-------------------------------------------
Bp_08986        -------MFVIVLCALV-------------------------------------------
Ac_LYZg1        PTFATSRPPFWNLVSSL-------------------------------------------
Ec_42267        TLAMMDSRLISLAVSLTCFL----------------------------------------
Cu_13863        ------------------------------------------------------------
Bg_LYZg6        -------MRLFIICALL-------------------------------------------
Bg_LYZg7        -------MRLFIICALL-------------------------------------------
Bp_08985        -------MRLFIICALL-------------------------------------------
Ac_LYZg2        -------MFIVLTLALL-------------------------------------------
Pa_37057        -------MYLVVLCALV-------------------------------------------
Pa_34965        -------MFLLVLCSVL-------------------------------------------
Pa_44383        -------MHLIILGAFL-------------------------------------------
Bg_LYZg4        -------MQVFILCALV-------------------------------------------
Bg_LYZg5        -------MQVFLLCAIV-------------------------------------------
Se_85121        ------------------------------------------------------------
Pm_29535        AQTARFTMNVLAVVTLLAIS----------------------------------------
My_59246        LVGQISTMNPLVIVTLLAIS----------------------------------------
Mg_gewl         ------MKTFFLLSAVI-------------------------------------------
Mg_gewl2        ------MKTFLLLSAVI-------------------------------------------
Lg_LYZg2        RPSCYNGRFVNGLCSSNYKCCVPNTPKPTK------------------------------
Ls_01764        GLEIGSLCAGDQVCCFTSNTCG--------------------------------------
Cm_LYZT3a       DHLQCTGQYQSYLCSGAANRRCCIPSRSSSG-----------------------------
Cm_LYZg2        VQRQCTGQYHSNLCSGAANRRCCIPSSTSSG-----------------------------
Ac_LYZg3        -------MILFLVLTSL-------------------------------------------
Lg_LYZg3        ------------------------------------------------------------
Hdd_lyzg        NFKLASLCSGSDVCCFSHDTC---------------------------------------
Hr_78614        SFMVASLCSGSNVCCFSHDTC---------------------------------------
Hdi_LysG1       GFMMASLCAGNDVCCFSHDSC---------------------------------------
Pm_gewl         ALQLSGLCSGSDVCCFTSDTCK--------------------------------------
Lg_LYZg1        ------------------------------------------------------------
Pc_09293        -----MIRWVFLFGVL--------------------------------------------
Pv_09293        SLQLGNLCSGSEVCCFSSNTC---------------------------------------


Cc_94995        ------------------------------------------------------------
Cc_98523        ------------------------------------------------------------
Cc_98591        ------------------------------------------------------------
Hs_LYG1         ------------------------------------------------------------
Hs_LYG2         ------------------------------------------------------------
Dg_27517        ------------------------------------------------------------
Xsp_46186       ------------------------------------------------------------
Myx_gewl        ------------------------------------------------------------
Myx_34660       ------------------------------------------------------------
Bf_LYZg6        ------------------------------------------------------------
Bf_LYZg3        ------------------------------------------------------------
La_79286        ------------------------------------------------------------
Bf_LYZg1        ------------------------------------------------------------
Bf_LYZg5        ------------------------------------------------------------
Bf_LYZg4        ------------------------------------------------------------
Bf_LYZg2        ------------------------------------------------------------
```

```
Ci_76087        ------------------------------------------------------------
Sc_LYZT3a       ------------------------------------------------------------
Cl_S3624        ------------------------------------------------------------
Cl_10499        PGSYHSGLC---------------------------------------------------
Tsp_Lys         ------------------------------------------------------------
Ta_63166        ------------------------------------------------------------
Pca_Gewl        ------------------------------------------------------------
Pyx_gewl        ------------------------------------------------------------
Arc_gewl        ------------------------------------------------------------
Sa_gewl         ------------------------------------------------------------
Hya_gewl        ------------------------------------------------------------
Cg_26612        ------------------------------------------------------------
Ca_78719        ------------------------------------------------------------
Se_82739        ------------------------------------------------------------
Oe_46487        VNMFFIHHTEMEHCDNTESCSSSMRIIQRFHMKDRGWNDIAYSFLIGGDGQVYEGRGWDR
Pc_57781        ------------------------------------------------------------
Pc_57861        ------------------------------------------------------------
Ls_08512        ------------------------------------------------------------
Ec_47861        ------------------------------------------------------------
Ech_17175       ------------------------------------------------------------
Bg_LYZg2        ------------------------------------------------------------
Bp_08972        ------------------------------------------------------------
Bt_15341        L-----------------------------------------------------------
Bt_43005        ------------------------------------------------------------
Bt_31685        ------------------------------------------------------------
Bg_LYZg1        ------------------------------------------------------------
Bp_05359        ------------------------------------------------------------
Bf_05346        ------------------------------------------------------------
Bg_LYZg3        ------------------------------------------------------------
Bp_08986        ------------------------------------------------------------
Ac_LYZg1        ------------------------------------------------------------
Ec_42267        ------------------------------------------------------------
Cu_13863        ------------------------------------------------------------
Bg_LYZg6        ------------------------------------------------------------
Bg_LYZg7        ------------------------------------------------------------
Bp_08985        ------------------------------------------------------------
Ac_LYZg2        ------------------------------------------------------------
Pa_37057        ------------------------------------------------------------
Pa_34965        ------------------------------------------------------------
Pa_44383        ------------------------------------------------------------
Bg_LYZg4        ------------------------------------------------------------
Bg_LYZg5        ------------------------------------------------------------
Se_85121        ------------------------------------------------------------
Pm_29535        ------------------------------------------------------------
My_59246        ------------------------------------------------------------
Mg_gewl         ------------------------------------------------------------
Mg_gewl2        ------------------------------------------------------------
Lg_LYZg2        ------------------------------------------------------------
Ls_01764        ------------------------------------------------------------
Cm_LYZT3a       ------------------------------------------------------------
Cm_LYZg2        ------------------------------------------------------------
Ac_LYZg3        ------------------------------------------------------------
Lg_LYZg3        ------------------------------------------------------------
Hdd_lyzg        ------------------------------------------------------------
Hr_78614        ------------------------------------------------------------
Hdi_LysG1       ------------------------------------------------------------
Pm_gewl         ------------------------------------------------------------
Lg_LYZg1        ------------------------------------------------------------
Pc_09293        ------------------------------------------------------------
Pv_09293        ------------------------------------------------------------


Cc_94995        ------------------------------------------------------------
Cc_98523        ------------------------------------------------------SPSWQP
Cc_98591        ------------------------------------------------------VKANAT
Hs_LYG1         ------------------------------------------------------DLSESS
Hs_LYG2         ------------------------------------------------------SYPFSH
Dg_27517        ------------------------------------------------------------
Xsp_46186       ------------------------------------------------------------
Myx_gewl        ------------------------------------------------------ISNDAG
Myx_34660       ------------------------------------------------------GVTATS
```

```
Bf_LYZg6        ------------------------------------------------------------
Bf_LYZg3        ------------------------------------------------------GEGGGG
La_79286        ------------------------------------------------------EGGFGG
Bf_LYZg1        ------------------------------------------------------AVAAAS
Bf_LYZg5        ------------------------------------------------------GGTSGT
Bf_LYZg4        ------------------------------------------------------------
Bf_LYZg2        ------------------------------------------------------------
Ci_76087        ------------------------------------------------------GVAIAY
Sc_LYZT3a       ------------------------------------------------------GGLIGG
Cl_S3624        ------------------------------------------------------STGSSS
Cl_10499        ------------------------------------------------------GGPSAR
Tsp_Lys         ------------------------------------------------------GYCPGP
Ta_63166        ------------------------------------------------------------
Pca_Gewl        ------------------------------------------------------AASSAA
Pyx_gewl        ------------------------------------------------------ATASAA
Arc_gewl        ------------------------------------------------------ATASAA
Sa_gewl         ------------------------------------------------------ATSTRA
Hya_gewl        ------------------------------------------------------STTTRA
Cg_26612        ------------------------------------------------------SLSAGY
Ca_78719        ------------------------------------------------------SLSAGY
Se_82739        ------------------------------------------------------GVVNAT
Oe_46487        VGSHTYRYDYVSLAVAFIGDFNDTMPGSIALSASQNLLTCGVHRNAIKTSYSLYGHRDVR
Pc_57781        ------------------------------------------------------SSALAA
Pc_57861        ------------------------------------------------------VSSALA
Ls_08512        ------------------------------------------------------ASSALA
Ec_47861        ------------------------------------------------------SGPVGN
Ech_17175       ------------------------------------------------------GGHVTG
Bg_LYZg2        ------------------------------------------------------SIPKPA
Bp_08972        ------------------------------------------------------SIPKPA
Bt_15341        ------------------------------------------------------SLATAA
Bt_43005        ------------------------------------------------------GLVTAA
Bt_31685        ------------------------------------------------------GLVTAA
Bg_LYZg1        ------------------------------------------------------GSTSAA
Bp_05359        ------------------------------------------------------GFTSAA
Bf_05346        ------------------------------------------------------GFTSAA
Bg_LYZg3        ------------------------------------------------------AYANAA
Bp_08986        ------------------------------------------------------AYANAA
Ac_LYZg1        ------------------------------------------------------------
Ec_42267        ------------------------------------------------------GFAAAA
Cu_13863        ------------------------------------------------------------
Bg_LYZg6        ------------------------------------------------------SLANAA
Bg_LYZg7        ------------------------------------------------------SLANAA
Bp_08985        ------------------------------------------------------SLANAA
Ac_LYZg2        ------------------------------------------------------ATADAA
Pa_37057        ------------------------------------------------------AYANAA
Pa_34965        ------------------------------------------------------AYAYAA
Pa_44383        ------------------------------------------------------AVANAA
Bg_LYZg4        ------------------------------------------------------AVSYAA
Bg_LYZg5        ------------------------------------------------------AVGYAA
Se_85121        ------------------------------------------------------------
Pm_29535        ------------------------------------------------------TSAWAA
My_59246        ------------------------------------------------------TGAWAA
Mg_gewl         ------------------------------------------------------FATDAA
Mg_gewl2        ------------------------------------------------------FATDAA
Lg_LYZg2        ------------------------------------------------------TTPPPV
Ls_01764        ------------------------------------------------------GSSSGG
Cm_LYZT3a       ------------------------------------------------------SSPSSG
Cm_LYZg2        ------------------------------------------------------SSHSSG
Ac_LYZg3        ------------------------------------------------------GVAYGT
Lg_LYZg3        ------------------------------------------------------------
Hdd_lyzg        ------------------------------------------------------GSSSGG
Hr_78614        ------------------------------------------------------GSSS--
Hdi_LysG1       ------------------------------------------------------GSSS--
Pm_gewl         ------------------------------------------------------GGSSSS
Lg_LYZg1        ------------------------------------------------------------
Pc_09293        ------------------------------------------------------AAAKGA
Pv_09293        ------------------------------------------------------GGGSSA


Cc_94995        ----------------------------------------------VSC-----------
Cc_98523        S---------------------------------------------RHP-----------
```

```
Cc_98591        P--------------------------------------------SRQP-----------
Hs_LYG1         N---------------------------------------------WGC-----------
Hs_LYG2         S-----------------------------------MKPHLHPRLYHGC-----------
Dg_27517        ------------------------------------------------------------
Xsp_46186       ------------------------------------------------------------
Myx_gewl        T---------------------------------------------KDL-----------
Myx_34660       A-----------------------------------------RNDAANP-----------
Bf_LYZg6        ------------------------------------------------------------
Bf_LYZg3        G-------------------------------------PAQEQQQDQDC-----------
La_79286        G-------------------------------------PSNQQQQDKDC-----------
Bf_LYZg1        G-----------------------------------------------N-----------
Bf_LYZg5        G---------------------------------------------SGS-----------
Bf_LYZg4        ------------------------------------------------------------
Bf_LYZg2        ---------------------------------------------INRN-----------
Ci_76087        T----------------------------------------------GC-----------
Sc_LYZT3a       G---------------------------------------------IGCSSTK-------
Cl_S3624        N---------------------------------------------TGC-----------
Cl_10499        RCCIASSTPSSASDSQCTVRGGKCQLDSIYCPGSYLSGLCGGPSSRRCCIASSTPSGGGQ
Tsp_Lys         S-------------------------------------------SVRCCVSSSGSGGSYP
Ta_63166        ------------------------------------------------------------
Pca_Gewl        A---------------------------------------------SNP-----------
Pyx_gewl        A---------------------------------------------SNP-----------
Arc_gewl        A---------------------------------------------SNP-----------
Sa_gewl         A---------------------------------------------SNP-----------
Hya_gewl        A---------------------------------------------SNP-----------
Cg_26612        N---------------------------------------------YSC-----------
Ca_78719        N---------------------------------------------YSC-----------
Se_82739        G--------------------------------------------NYTC-----------
Oe_46487        GTSCPGYTLYNAIRKWEHYNVTTDKDGPCLEKGGLCGPTYLPCKGGVNC-----------
Pc_57781        T----------------------------------------------NC-----------
Pc_57861        A---------------------------------------------NTC-----------
Ls_08512        A---------------------------------------------TSC-----------
Ec_47861        G---------------------------------------------RTC-----------
Ech_17175       G---------------------------------------------RTC-----------
Bg_LYZg2        P---------------------------------------------RTC-----------
Bp_08972        P---------------------------------------------RTC-----------
Bt_15341        K---------------------------------------------RTC-----------
Bt_43005        S---------------------------------------------RTC-----------
Bt_31685        S---------------------------------------------RTC-----------
Bg_LYZg1        ----------------------------------------------RTC-----------
Bp_05359        ----------------------------------------------RTC-----------
Bf_05346        ----------------------------------------------RTC-----------
Bg_LYZg3        N---------------------------------------------RLC-----------
Bp_08986        N---------------------------------------------RLC-----------
Ac_LYZg1        ----------------------------------------------RTC-----------
Ec_42267        S---------------------------------------------RTC-----------
Cu_13863        N---------------------------------------------RLC-----------
Bg_LYZg6        N---------------------------------------------RLC-----------
Bg_LYZg7        N---------------------------------------------RLC-----------
Bp_08985        N---------------------------------------------RLC-----------
Ac_LYZg2        S---------------------------------------------RTC-----------
Pa_37057        N---------------------------------------------RLC-----------
Pa_34965        P---------------------------------------------RTC-----------
Pa_44383        S---------------------------------------------RTC-----------
Bg_LYZg4        P---------------------------------------------RTC-----------
Bg_LYZg5        P---------------------------------------------RTC-----------
Se_85121        -----------------------------------------------DC-----------
Pm_29535        S---------------------------------------------YTC-----------
My_59246        S---------------------------------------------YTC-----------
Mg_gewl         N---------------------------------------------YNC-----------
Mg_gewl2        N---------------------------------------------YNC-----------
Lg_LYZg2        S---------------------------------------------YQC-----------
Ls_01764        N---------------------------------------------YNC-----------
Cm_LYZT3a       N---------------------------------------------YNC-----------
Cm_LYZg2        G---------------------------------------------YNC-----------
Ac_LYZg3        N---------------------------------------------YKC-----------
Lg_LYZg3        -----------------------------------------------MC-----------
Hdd_lyzg        N---------------------------------------------YNC-----------
Hr_78614        N---------------------------------------------YNC-----------
Hdi_LysG1       K---------------------------------------------YNC-----------
Pm_gewl         G---------------------------------------------YNC-----------
```

```
Lg_LYZg1        ------------------------------------------------C-----------
Pc_09293        G---------------------------------------------YNC-----------
Pv_09293        G---------------------------------------------YNC-----------


Cc_94995        -----SNDLRGLQNTWHPSFGAKIYSLTDGWEYASATGARFG----GVAKSERDVSWDI-
Cc_98523        -----YAD--TIYPL------------TAGWEQATATGARYG----GVSKSEYRVSSDV-
Cc_98591        -----YAD--TIYPL------------TAGWEQTIATGARYG----GVSKSEYRVSSDV-
Hs_LYG1         -----YGNIQSLDTP------------GASCGIGRRHGLNYC----GVRASERLAEIDM-
Hs_LYG2         -----YGDIMTMKTS------------GA---TCDANSVMNC----GIRGSEMFAEMDL-
Dg_27517        ------MSVRNIQTA------------GASTRTASQDRLPA-----GIGSSQKMAERDG-
Xsp_46186       ------MSVRNISTT------------GASAATARQDRLPA-----GVASSEAMAERDG-
Myx_gewl        -----NAILSRYKPT------------GASQRTASQDGLTA-----GVNASTKMAQTDA-
Myx_34660       -----NAILSQYTPT------------GASARTARQDG-HQA----GVDASKKMAQADL-
Bf_LYZg6        -----YGDLTKVDAT------------GASDATAKQDKLKIS----GEAASQKMAKTDK-
Bf_LYZg3        -----FGDVDKVDTT------------GASQATAQQDGLSTT----GVAASNALAQTDL-
La_79286        -----HGDVGKVDTK------------GASSKTAGQDANAKG--LTGVAASNKLAQNDL-
Bf_LYZg1        -----YGNIMAVDTT------------GASAQTASQDGIGYG----GTSASQQMARTDL-
Bf_LYZg5        -----YGNVMEIDTT------------GASSQTASQDNIWYS----GTSASHQLASNDL-
Bf_LYZg4        --------IMNVDTT------------GASQTTANQDGLSYN----GVPASQQLARNDL-
Bf_LYZg2        -----YGNILAVDTT------------GASADTASQDGLGYTALFIGVAASHQMASTDL-
Ci_76087        -----YGNIYNTDTT------------GCSSQTSSQDNLGYS----GISASRQMAATDL-
Sc_LYZT3a       -----YGNIRNVDTT------------GASYATASQDGLS-G----GVSSSHKMAQNDK-
Cl_S3624        -----YGDIEKVDTT------------GASSATASQDNLYYS----GVPASRKMAEYDL-
Cl_10499        CISCTLGDIADLNPT------------GASQRTARQDRLNYA----GVRASEKLANTDL-
Tsp_Lys         TK---YGDFMRINPS------------GASSATARQDGLSYS----GVAASNKLASNDY-
Ta_63166        ---------MRINPS------------GASSATARQDGLSYS----GVAASNKLASNDY-
Pca_Gewl        -----NAILSKYQPT------------GASSATARQDGLQ-G----GVAASRKMAQTDL-
Pyx_gewl        -----NAILSKYQPT------------GASSATARQDGLQ-G----GVAASRKMAQTDL-
Arc_gewl        -----NAILSKYQPT------------GASSATARQDGLQ-G----GVAASRKMAQTDL-
Sa_gewl         -----NAILSKYQPT------------GASAATARQDGLP-A----GVASSRKMARTDL-
Hya_gewl        -----NAILSKYQPT------------GASSATARQDGLP-G----GVASSQKMARTDL-
Cg_26612        -----FGDFTLLHPT------------GK----------SNG----GVQSSQLDVSKTY-
Ca_78719        -----FGDFTLLHPT------------GK----------SNG----GVQSSQLDVSKTY-
Se_82739        -----YGNFMSLTPT------------GA----------KRG----GVSTSQYDVSYLYD
Oe_46487        -----YGDFMLLRPT------------GR----------KSG----GVQESRRDVDYHF-
Pc_57781        -----YGDIRNLRPT------------GK----------SNG----GVSASQAAVNARI-
Pc_57861        -----HGNIDNLTPT------------GM----------HSG----GVTASQSAVRAHL-
Ls_08512        -----YGNVNNLRPT------------GK----------HSG----GTSASHSAVAAHL-
Ec_47861        -----YGDINDLQPS------------GE----------ASG----GVPGSQADVRMDI-
Ech_17175       -----HGDIAQLRPT------------GE----------AGR----GVAGSQADVITDL-
Bg_LYZg2        -----YGDVNDLKPT------------GR----------KKG----GVAASNKMIQADM-
Bp_08972        -----YGDVNALNPT------------GR----------KKG----GVAASNRMIQADL-
Bt_15341        -----YGDIKHLSPT------------GR----------KKG----GVAASNNNVKADL-
Bt_43005        -----YGDINNLTPT------------GR----------KNG----GVQTSNNEARVDI-
Bt_31685        -----YGDINNLTPT------------GR----------KNG----GVPTSNNEARVDM-
Bg_LYZg1        -----YGDINNLSPT------------GR----------KVG----GVTKSNSEVQTDL-
Bp_05359        -----YGDINNLSPT------------GR----------KVG----GVTKSNSEVQTDL-
Bf_05346        -----YGDINNLSPT------------GR----------KFE----GVTKSNSEVQTDL-
Bg_LYZg3        -----YGDIMKLTPH------------GK----------ASG----GVTASNSIVSSDL-
Bp_08986        -----YGDVMKLTPH------------GK----------ASG----GVTASNSIVSSDL-
Ac_LYZg1        -----HGNVMDLSPT------------GQ----------KSG----GVSASNSDAQADI-
Ec_42267        -----HGSVARLSPT------------GR----------LNG----GVAASEREVRYDL-
Cu_13863        -----HGNIDHLNPS------------GQ----------ANG----GVAASNNDAQYDM-
Bg_LYZg6        -----NGDVMTLHPT------------GK----------ASG----GVAASHAEVQHDV-
Bg_LYZg7        -----NGDVMTLHPT------------GK----------ASG----GVAASHAEVQHDV-
Bp_08985        -----NGDVMTLHPT------------GK----------ASG----GVAASHAEVQHDV-
Ac_LYZg2        -----HGDLNSLHPK------------GQ----------SNG----GVAASNRDAQHDL-
Pa_37057        -----HGDIDQLHPT------------GK----------HTG----GVAGSNAEVQHDL-
Pa_34965        -----HGDVMQLHPT------------GK----------HTG----GVAASNAEVQHDL-
Pa_44383        -----HGDINHLHPS------------GK----------ANG----GVAASNADVQHDV-
Bg_LYZg4        -----HGDINNLHPT------------GK----------ASG----GVAGSNAEVTHDI-
Bg_LYZg5        -----HGDINNLHPK------------GK----------ASG----GVAASEADVAYDI-
Se_85121        -----FGDVTKLTPS------------GR----------KNG----GIPASINEITPDL-
Pm_29535        -----HGDVRRLHPT------------GE----------HNG----GVAASNRDVDYDY-
My_59246        -----HGDVRRLHPT------------GE----------HNG----GVAASNHDVDYDL-
Mg_gewl         -----HGDVTQLHPT------------GM--------GSAYG----GMAGSHQAIDQDI-
Mg_gewl2        -----HGDVTQLHPT------------GM--------GSAYG----GMAGSHQAIDQDI-
Lg_LYZg2        -----YGDFMKLNPT------------GAGKLTSGQDWLKYT----GVRASNKMLDTDL-
Ls_01764        -----FGNFMNLHPT------------GSSAQTARQDGISGG----GVAASQRMVANDY-
Cm_LYZT3a       -----YGNFMNLHPT------------GASAQTARQDRIRQG----GVSASQRMVANDI-
```

```
Cm_LYZg2        -----FGNFMNLHPT------------GASTQTARQDRIGHG----GVSASQRMVSNDI-
Ac_LYZg3        -----HGNVMVLTVS------------GASSSTARQDGLSTG----GLQSSYKMADNDY-
Lg_LYZg3        -----HGDFMKLMPK------------GADQRTARQDNLAYA----GVRASNKLVDNDL-
Hdd_lyzg        -----YGDVMKLHPA------------GASARTSS--GLGYS----GVQASNHMVDQDY-
Hr_78614        -----YGDVMKLHPT------------GASTRTSS--GLGYS----GVQASNHMVDQDY-
Hdi_LysG1       -----YGDVMKLRPT------------GASSRTSS--GLGYS----GVQASNHMVDQDY-
Pm_gewl         -----HGDVMKLHPT------------GASSRTAAQDGIHYG----GAQASYKMLDNDL-
Lg_LYZg1        -----HGDVMKLHPT------------GASSRTASQDGIHYG----GAQASYKMLDNDL-
Pc_09293        -----YGNVMSIHPT------------GASARTASQDGIHYG----GVQASYKMVDNDL-
Pv_09293        -----YGNVMSIHPT------------GASSRTASQDGINYG----GAQASYKMVDNDL-
                                                             *   *

Cc_94995        TRLKEVKCN-----------IVAAGRLTGIDPAILAAIASRESGAGLRL-NSRGYGNSFS
Cc_98523        WRLEKVKSK-----------IVAAGRLTGIDPAILAAIASRGSAAGYQL-NSRGYSYHST
Cc_98591        WRLEKVKCK-----------IVAAGRLTGIDPAILAAIASRGSEAGYQL-NSRGYNYHSA
Hs_LYG1         PYLLKYQPM-----------MQTIGQKYCMDPAVIAGVLSRKSPGDKILVNM---GDRTS
Hs_LYG2         RAIKPYQTL-----------IKEVGQRHCVDPAVIAAIISRESHGGSVL--QDGWDHRGL
Dg_27517        RYIQAHKET-----------IISVGRELSVEPALIAAIISRESRGGTAIEQTGGWGDHGQ
Xsp_46186       RYIQAYKEI-----------IHEVGRELNIGPSLIAAIISRESRGGTAL--KEGWGDNGN
Myx_gewl        ARLKKYAAE-----------FEAAGKKYDLPPALLAAIASRESRAGAAL-DSRGLGDNGN
Myx_34660       PRILPYKAA-----------IEAAAQKHGVPPALLAAIASRESRGGAAL-DRTGHGDAGN
Bf_LYZg6        KNVDKYKDI-----------IKKVAKEKNIDPAIIAAIISRETRGNTGI-LENGWGDGGN
Bf_LYZg3        NRLNKYKTQ-----------INAVSKATGMDAAIIAAIISRESRAGNAL--QNGYGDHGN
La_79286        NRLKQFKEK-----------INQVSKETGMDAAIIAAIISRESRGGAAL-DSNHHGDNGN
Bf_LYZg1        NRLNTYKSK-----------IYNAASAKNMDPAVIAAIISRESRAGAAL-ASDGTGDNGN
Bf_LYZg5        SRLNNYKTQ-----------IFEAANARNMDAAVIAAIISRESRAGAAL-ASDGTGDHGN
Bf_LYZg4        SRLNNYKSQ-----------IEEAAADKNMDPAVIAATISRESRAGNAL-DTNGYGDHGN
Bf_LYZg2        SRLNNYKSQ-----------ILQAASAKNMDPAVIAAIISRETRAGAAL-APDGTGDNGN
Ci_76087        SRMNQYKSN-----------IENAGRQLCMDPSVIAGIISRETRAGATI-GSDGYGADGH
Sc_LYZT3a       NAMLQYKSL-----------IQTAANDLCMDAAVIAGIISRETRAGKVL-GSDGFGYDGH
Cl_S3624        TAMSQYKTK-----------IVQAGEKLCADPAIIAGIISRETRAGKVL-GSDGFGSDGH
Cl_10499        MRMKQYKNL-----------IAKVAKDLNFDGAIIAAIISRESRAGAAL-DSKGYGDHGN
Tsp_Lys         NRCLRYKSQ-----------FQSASSSTQIPVGLITAIASRESRCGGAL-DSNGYGDHGN
Ta_63166        NRCLRYKSQ-----------FQSASSSTQIPVGLITAIASRESRCGGAL-DSNGYGDHGN
Pca_Gewl        PKIKKYADE-----------FAAAGKKYNLPPALLAAIASRESRGGSAL-DSRGFGDHGN
Pyx_gewl        PKIKKYADE-----------FAAAGKKYNLPPALLAAIASRESRGGSAL-DSRGFGDHGN
Arc_gewl        PKIKKYADE-----------FAAAGKKYNLPPALLAAIASRESRGGSAL-DSRGFGDHGN
Sa_gewl         PKIKQYAGE-----------FAAAGKKHDLPPALLAAIASRESRGGSAL-DSRGYGDHGN
Hya_gewl        TKMKKYADE-----------FAAAGKKHNLPPALLAAIASRESRGGSAL-DSRGFGDHGN
Cg_26612        NLAHSYKAC-----------FEQIGQFTCIHPAIIAAVASRETNVGADITVSRGWNAGHF
Ca_78719        NLAHSYKAC-----------FEQIGQFTCIHPAIIAAVASRETNVGADITVSRGWNAGHF
Se_82739        SRADVYKSC-----------FEFVGRDLCIHPAVIAGMASRETNVGYDIRNTNGWGDHGN
Oe_46487        SLANEFHSC-----------FVQVGNEVCLHPAVIAGVASRESNFGYSL-TPDGWGDHHH
Pc_57781        KELESLRSC-----------YDQAADKYCIQASVIGALASRESDGGASL-TPDGYGDSRK
Pc_57861        TTLNGLKTC-----------YEQVAATHCIEASVIGGLASRESNGGDSL-TAAGYGDNGH
Ls_08512        HTLTAMKSC-----------YEQVANSNCIEASVIAALASRESNGGDSL-TSAGYGDGGH
Ec_47861        PELKKRWAF-----------YQAVADQNCVQASLIAALASRESRGGLLLYKTDGYGDHGR
Ech_17175       PELKKRWAF-----------YQTVADLNCVQASLIAALASRESRGGALLYKTNGYGDNGR
Bg_LYZg2        ASLTKYREC-----------FQKVADKFCIQASILGAIASRESRGGALLDKTKGFGDKGN
Bp_08972        ASLTKYREC-----------FQKVADKFCIQASILGAIASRESRGGALLYKTKGFGDKGN
Bt_15341        QSLNKYKSC-----------YQNVADSVCIQASVIAAIASRESRGGSWINRTRGYGDRGR
Bt_43005        PYLNRYRDC-----------YQSAADKYCIQASVIAAIASRESRGGSYIEKTNGYGDSGK
Bt_31685        PYLNRYRDC-----------YQSAADKYCIQASVIAAIASRESRGGSYIEKTNGYGDSGK
Bg_LYZg1        TYLNRYRSC-----------YQTAADKLCIQASVIAAVASRESRGGSLLEKTNGYGDGGK
Bp_05359        TYLNRYRSC-----------YQTAADKLCIQASVIAAVASRESRGGSLLEKTNGYGDGGK
Bf_05346        TYLNRYRSC-----------YQTAADKLCIQASVIAAVASRESRGGSLLEKTNGYGDGGK
Bg_LYZg3        AALTAHKNC-----------YDASADRNCIQASVIAALASRESNGGSAL--RNGYGDGGK
Bp_08986        AALNAHKNC-----------YDASADRNCIQASVIAALASRESNGGKAL--QNGYGDGGK
Ac_LYZg1        TYLNKYKTC-----------FQKVAVSQCIQASLIAALASRESRGGSLL--KDGYGDHKK
Ec_42267        PELEKRRTC-----------YQQVADENCLQASVIAALASRESRGGKLLYSTGGYGDGGR
Cu_13863        NDLNAHRDC-----------YAASADKNCIQASLLAAIASRESRGGRLLYATGGYGDNNN
Bg_LYZg6        TALEKHRHC-----------YQASADNNCIQASVIAAVASRESRGGTLLVATHGYGDLGH
Bg_LYZg7        TALEKHRHC-----------YQASADNNCIQASVIAAVASRESRGGTLLVATHGYGDLGH
Bp_08985        TALEKHRHC-----------YQASADNNCIQASVIAAVASRESRGGTLLVATHGYGDLGH
Ac_LYZg2        PYLEKHRSC-----------YQQAADHNCIQASVIAALASRESRGGSLLESTGGYGDPGA
Pa_37057        PALNNHRSC-----------YQQSADTNCIQASVIAALASRESRGGSLLVSTGGWGDNHH
Pa_34965        PALNNHRHC-----------YQATADNNCIQASVIAAIASRESRGGSLLVSTGGYGDHGA
Pa_44383        PALNQHRNCYQQSADHNCKSYQQSADHNCIQASVIAAIASRESRGGTLLVATGGYGDFGH
Bg_LYZg4        PALEKHRNC-----------YQASADKNCIQASVIAAIASRETRGGTLLVATGGWGDNHN
Bg_LYZg5        PALERHRNC-----------YQASADRNCIQASVLAAIASRESRGGTLLVATGGWGDNHN
Se_85121        KKLTEKKSC-----------YVSAGENNCVHPAVIAAIASRESHAGRLLINTNGWGDNDN
```

```
Pm_29535            NDLRDKKSC-----------YDQAGAAYCIQPAVIAALASRESRGGRLLHSTGGWGDHHH
My_59246            HDLNNKKSC-----------YYASGAAHCIQPSVIAALASRESRGGRLLTSTGGWGDHHH
Mg_gewl             AEINKRKSC-----------YVQAGAANCIHPAVIAGLASRESRAGKLLYSTSGWGDHHN
Mg_gewl2            AEINKRKSC-----------YVQAGAANCIHPAVIAGLASRESRAGKLLYSTSGWGDHHN
Lg_LYZg2            AELNKRKQC-----------YIRAGRNSCIHPAIIAAIASRESRGGRLLYASNGLGDKKK
Ls_01764            AELNKRKSC-----------YVTAGNKNCVHPAVIAGIASRETRGGSVIARTGGWGDNHH
Cm_LYZT3a           RELRKRKSC-----------YIQAGNNNCVHPAVIAGIASRETRGGAIIERTGGWGDNHN
Cm_LYZg2            NELNNRKSC-----------YIQAGNNNCVHPAVIAGIASRESRGGAAIKRTGGWGDNHN
Ac_LYZg3            SELNKRKTC-----------YYQAGKNNCIQPAVVAALASRESRGGKLLYRTKGYGDGYH
Lg_LYZg3            AELNKRKDC-----------YVQAGKNHCIHPAVIAAIASRETRGGKLLYSTNGYGDGGR
Hdd_lyzg            AELSKRKSC-----------YVRAGANNCIHPAVVAGVASRETRGGKLLYSTGGWGDHHH
Hr_78614            AELNKRKSC-----------YVRAGANNCIHPAVVAGVASRETRGGKLLYSTGGWGDHHH
Hdi_LysG1           AELNKRKSC-----------YVRAGANNCIHPAVVAGVASRETRGGKLLYSTGGWGDHHH
Pm_gewl             AELNKRKQC-----------YVQAGKNSCVHPAVIAAVASRETRGGKLLYRTGGWGDHHR
Lg_LYZg1            AELNKRKSC-----------YVQAGTNNCVHPAVIAAVASRETRGGKLLYRTGGWGDNHR
Pc_09293            GELNKRKSC-----------YVQAGNNNCLHPAVIAAVASRETRAGKLLYSTGGWGDHHR
Pv_09293            AELNKRKSC-----------YVQAGKNNCLHPAVIAAVASRETRGGKLLYSTGGWGDNHR
                                            .       .:: .  ** :  .  :      .

Cc_94995            TKYGYMQLDTRRYYV--------DTSEGPNGLAYFDQAAE-ELRSAIIYVETKRPYWEKP
Cc_98523            TKYGYMQLSTTRYYV--------DTSGGPSGQAHFDQAAK-VLKSALEYVEDSRPYWQKA
Cc_98591            TKYGYMQLSTTRNHV--------VTSGGPSGQPHFDQAAK-VLRSALEYVEDHRSYWQKA
Hs_LYG1             -----MVQDPGSQA----------PTSWISESQVSQTTE-VLTTRIKEIQRRFPTWTPD
Hs_LYG2             K-FGLMQLDKQTYHP----------VGAWDSKEHLSQATG-ILTERIKAIQKKFPTWSVA
Dg_27517            A-FGLMQIDKNWHTP----------QGGWNSREHIYQATN-ILRYFIDA--KNNPFSDPD
Xsp_46186           A-FGLMQIDKRWHTP----------RGAWNSKEHVGQATN-ILCDFIRSSDNPFRHHP-D
Myx_gewl            G-FGLMQVDKRYHKP----------EGGPYSANHIDQAAG-ILKGFLNDVKKAHPDWSPE
Myx_34660           G-FGLMQVDHRYHST----------KGGPTSAAHIDQAAG-ILKGYLNDVKKAHPDWPEA
Bf_LYZg6            G-WGLMQVDKNHHTP----------VGGATSIEHIRQGTD-ILIQSIKDIQKKFPKWTKE
Bf_LYZg3            G-FGLMQIDRRYHTT----------QGAWDSTEHLTQAVG-ILQGFIQGVSNCHPSWNSE
La_79286            G-FGLMQIDKRFHTT----------QGAWNSAEHIKQGVG-ILQGMINGVRDCHPSWSDE
Bf_LYZg1            G-YGLMQVDIRYHTP----------QGGPYTTTHIKQGTQ-ILIDTINCVKRNHPGWSTE
Bf_LYZg5            G-YGLMQVDIRYHTP----------QGGPYSTTHMKQGTQ-ILIDTINCVKRNHPDWTLN
Bf_LYZg4            G-FGLMQVDKRYHTP----------VGGPYSTEHIKQGTQ-ILIDTINCVKNRHSDWTAE
Bf_LYZg2            G-FGLMQVDYRYHTP----------AGGPYSTEHMMQGTQ-ILIDTINCVRQRHPGWTAE
Ci_76087            G-YGLMQVDDRYHTI----------QGGPTSLDHILQGTG-ILISNIGQISSKFPSWDQN
Sc_LYZT3a           G-YGLMQVDDRYHTL----------QGGPYSLAHIKQGTG-ILIDMINGVKNKHPSWSQD
Cl_S3624            G-YGLMQVDDRYHTL----------KGGPYSLEHIEQGTG-ILIDMINGVEVKHRDWTQD
Cl_10499            G-YGLMQVDKRHHHR---------LKGGPYSEEHIRHGTE-ILISFIRKVQARFPSWTRA
Tsp_Lys             G-YGLMQVDKRYHSL----------QGGPYSSTHISQATN-ILISSINGVANNHRSWTKE
Ta_63166            G-YGLMQVDKRYHSL----------QGGPYSSTHISQATN-ILISSINGVANNHRSWTKE
Pca_Gewl            G-FGLMQVDKRFHSL----------KGGPFSAAHIDQAAG-ILKSYQNEIKARHPDWPPE
Pyx_gewl            G-FGLMQVDKRFHSL----------KGGPFSAAHIDQAAG-ILKSYQNQIKARHPDWPPE
Arc_gewl            G-FGLMQVDKRFHSL----------KGGPFSAAHIDQAAG-ILKSYQNQIKARHPDWPPE
Sa_gewl             G-FGLMQVDKRYHNP----------KGGPTSAAHIDQAAG-ILKSYHNQVKAKHPDWPPE
Hya_gewl            G-FGLMQVDKRFHSP----------KGGPFSAAHIDQAAG-ILKSYHNQIKAKHPDWPPE
Cg_26612            E-YGMLPCDTRSCLVCHKNVGLTCTSYPWNTCDHALMMAQYVLLPFVKSIQTKFPSWLSE
Ca_78719            E-YGMLPCDTRSCLVCHKNVGLTCTSYPWNTCDHALMMAQYVLLPFVKSIQTKFPSWLSE
Se_82739            G-YGILQCDIRHCPV--CNYNLTCTSYPYNSCQHINMMIKYVLIPNINSMKTKFPEWTPE
Oe_46487            A-YGMMQCDVQMCPV--CRYGLSCTKYRFDSCDHIRMMSRYVLVPYIQAVKAKFPTWLPQ
Pc_57781            A-WGVLQCDLSKSKL-------PCLDCGPRTCCHIDMMVGKVLIPFIKEVSTKFRGWSAE
Pc_57861            A-WGILQCDLTHSGL-------PCRECGARTCCHVEMMVGRLLIPYISQVSAKHPSWSLE
Ls_08512            A-WGILQCDLTHSGL-------PCKNCGARTCCHVEMMVKSLLVPYIRQVGSRHSSWSTE
Ec_47861            A-YGILQCDLIHSGL-------NCLACVWYSQCHIQMMVSKILVPYIQQIQRKFPTWTSS
Ech_17175           A-YGILQCDLQHSGL-------DCLACVWYSQCHIQMMVSQLLVPYIQQVQRKFPTWTSS
Bg_LYZg2            A-WGIMQCDIRYSGL-------NCTSVPWNSCAHIEMMTGQLIIPFMKQVKKKHRKWPAA
Bp_08972            A-WGIMQCDIRYSGL-------NCTSVPWNSCAHIEMMTGQLIIPFMKQVKKKHQKWPAA
Bt_15341            A-WGIMQCDIKHSGL-------PCTSVPWNSCAHIEMMTKKLLIPFIKQVQKKFKKWPSY
Bt_43005            A-YGIMQCDIAKSSL-------PCKSVSWNSCAHIEMMTGRALIPFIKQIQQKKPTWSAE
Bt_31685            A-YGIMQCDIAKSSL-------PCKSVPWDSCAHIEMMTGRALIPFIKQIQQKKPTWPAE
Bg_LYZg1            A-YGIMQCDIKNSGL-------PCTSVSWNSCAHIEMMTG-ALIAKINEVKRKFPNWPAE
Bp_05359            A-YGIMQCDIKKSGL-------PCTSVSWNSCAHIQMMTG-ALIAKINEVKKKFPDWPAE
Bf_05346            A-YGIMQCDIKKSGL-------PCTSVSWNSCAHIQMMTG-ALITKINEVKKKFPKWPAE
Bg_LYZg3            A-WGILQCDIVHSGQ-------PCTSVPWNSCEHIEMMVHNILLANIHTIQRMHPTWPPE
Bp_08986            A-WGILQCDIVHSGQ-------PCTSVAWNSCEHIEMMVHNILLANIHTIQRMHPTWPPE
Ac_LYZg1            A-WGILQCDIKKSGL-------NCKSCGWDSCCHVEMMVRDILVPNINSVKRKFPSWTDA
Ec_42267            A-WGILQCDRYTSGM-------NCMTCEWDSCCHIRMMVSQLLVPNIDAVRRKHPSWSID
Cu_13863            A-WGIMQCDLRYSGL-------NCKQCPWDSCCHIEMLTSQTLVPFINQIHNKFPSWSQD
Bg_LYZg6            A-WGIMQCDTRYSGL-------PCTSVPWDSCEHIEMMVHRMLVPNVHSLHTKHPAWPMS
Bg_LYZg7            A-WGIMQCDTRYSGL-------PCTSVPWDSCEHIEMMVHRMLVPNVHSLHTKHPAWPMS
Bp_08985            A-WGIMQCDTRYSGL-------PCTSVPWDSCEHIEMMVHRMLVPNVHSLHTKHPAWPMS
```

```
Ac_LYZg2          A-WGILQCDIRHSGL-------NCKSCDWDSCCHINMMVRDLLVPYINQVHRKHPSWTSA
Pa_37057          A-WGILQCDVANSGL-------PCTSVPWDSCAHIEMMVHRLLVPYINQVKAKHPDWSQH
Pa_34965          A-WGIMQCDITHSGL-------PCKSVPWDSCEHIEMMVHRLLVPYINQVHAKHPSWSQH
Pa_44383          A-WGIMQCDIANSGL-------PCTSVPWDSCAHIEMLVNRLLVPYVNQVHNKFPSWSID
Bg_LYZg4          A-WGIMQCDVRYSGL-------PCTSVPWDSCEHIEMLVNRLLVPYVTQVHNKFPSWSLD
Bg_LYZg5          A-WGIMQCDVRYSGL-------PCTSVPWDSCEHIEMLVNRLLVPYVNQVHNKFPSWSMD
Se_85121          A-YGTMQCDVRHCPVCDKTTGQNCTTYNWDSCEHINMMTKFTLVKFIKEVQKKHPNWALE
Pm_29535          A-YGILQCDIRYHS---------CTQHAWNSCAHISQMVHEVLVPYINQVARKHPTWSSE
My_59246          A-YGILQCDIRYHS---------CQKYAWDSCEHIEQMVSEVLVPYVNQVAHKHPTWSRD
Mg_gewl           A-YGIMQCDINANPL--HSIHKTCTSYHWDSCAHINAMTAHVLVPNIQGVKRKHHSWSDA
Mg_gewl2          A-YGIMQCDINANPL--HSIHKTCTSYHWDSCAHINAMTAHVLVPNIQGVKRKHHSWSDA
Lg_LYZg2          A-WGIMQCDLHKSGQ-------DCKKYKWDSCDHINHMTQTVLLPFVKTIKRNHPSWAAK
Ls_01764          A-YGIMQCDGGASGL-----GSTCTKYHWDSCEHIDMMVRILLVPYIKTIQHKHSSWAPE
Cm_LYZT3a         A-YGIMQCDGGASGL----GEEVCTRYLWNSCEHINMMVEILLVPYIRTIQSKHPSWAPE
Cm_LYZg2          A-YGIMQCDGGASGL-----GRRCTRYAWDSCEHINMMVEILLVPYIRTMQSKHPSWARE
Ac_LYZg3          A-YGILQCDGGASGL-----HSKCTQYAWDSCAHIDFMVKTLLVPYINQVKAKHPTWTQD
Lg_LYZg3          A-YGIMQVYI-------------CKKYPWDSCEHINQLTDIILLNYVNQMKTKHPSWPAH
Hdd_lyzg          A-WGIMQCDVFASGL-----GSTCQKYAWDSCDHIDQMTRIILVPYIKQVKAKHPTWSPE
Hr_78614          A-WGIMQCDVYASGL-----GSTCERYSWDSCDHIDQMTRVILVPYIKQVKAKHPTWSPE
Hdi_LysG1         A-WGIMQCDVYASGL-----GSTCERYAWDSCDHIDQMTRIILVPYIKQVKAKHPTWSPE
Pm_gewl           A-WGIMQCDLQASGL-----GSRCTQHGWDSCGHIDMMTKVILVPYMNQVKSRHSSWTAE
Lg_LYZg1          A-WGIMQCDLHASGL-----GSQCTKYGWDSCAHIDMMTRVILVPYMDQVKRKHPTWSAE
Pc_09293          A-WGIMQCDLHASGL-----GSQCTKYGWDSCAHIDMMTRVILVPYIKQVRSKHPTWSAE
Pv_09293          A-WGIMQCDLHASGL-----GSQCTKYGWDSCAHIDMMTRVILVPYIKQVRSKHPTWSAE
                       :                                   :

Cc_94995          MQIKGGIAAYDVGINNYSV-SAASTIDIGTTNHDYSNDVLARAKYL-RSKG---------
Cc_98523          MQVKAGIAAYDVGVQNYII-PSTIAIDTETTDKDYSNDVLARAKYLRRKRI---------
Cc_98591          MQVKAGIAAYDVGVHNYII-PSTSSIDMQTSNKDYSNDVLARAKYL-RRKG---------
Hs_LYG1           QYLRGGLCAYSGGAGY--V-RSSQDLSC-----DFCNDVLARAKYL-KRHG---------
Hs_LYG2           QHLKGGLSAFKSGIEA--I-ATPSDID-----NDFVNDIIARAKFY-KRQS---------
Dg_27517          QRLKAAIAAYNKGFQG--MNAGYNNIDAHTTGGDYSDDVVARAQYF-QRNG---------
Xsp_46186         QGLKAAIAAYNAGFKR--MSKNYNEIDNHTTGQDYSNDVVARAQYF-ERNG---------
Myx_gewl          QQLRGAVAAYNSGPKN--V-RTIQNMDVGTTGNDYSNDVWARAQALAKDFG---------
Myx_34660         QQLRGAVAAYNFGPKN--V-KTQGGMDQGTTGNDYSNDVWARAQALAPSFG---------
Bf_LYZg6          QQLKGGISAYNAGTGN--V-QTYENMDVGTTGNDYANDVVERAKWY-NQNG---------
Bf_LYZg3          EVLKGGISAYNAGVKN--V-RSYDRMDVGTTGDDYANDVIARAKYL-KTKG---------
La_79286          QVLKGGISAYNAGVRN--V-RNYDRMDVGTTGDDYANDVIARAQYL-KQKG---------
Bf_LYZg1          MALKGGISGYNAGCGN--V-QTYNGMDIGTTGDDYGNDVVARAQWL-KRNG---------
Bf_LYZg5          MALKGGISGYNAGCGN--V-QTYAGMDGGTTGEDYANDVVARAQWL-KGQG---------
Bf_LYZg4          MALKGGISGYNAGCGN--V-QTYAGMNVGTTGGDYANDVVARAQWL-RGQG---------
Bf_LYZg2          MALKGGISGYNAGCGN--V-QTYAGMDVNTTGDDYANDVVARAQWL-KAHG---------
Ci_76087          MDLKGGICAYNIGVGG--V-WSYDNMDVGTTGDDYSSDVVARAQYY-KDNG---------
Sc_LYZT3a         MALKGGISAYNAGVSN--V-DSYNNMDVGTTGNDYANDVVARSQWY-DDNG---------
Cl_S3624          MALKGGICAYNSGVSN--V-QTYENMDVGTTGNDYSNDVTARSQYY-KENG---------
Cl_10499          RQLQGGVAAYNFGLDN--V-RTWTRLDIGTTGNDYSSDVIARAKYF-RTQG---------
Tsp_Lys           QQMQGGVAAYNFGVGN--V-QSIGGMDIGTTGNDYSNDVVARAQWF-HNNG---------
Ta_63166          QQMQGGVAAYNFGVSN--V-QSIGGMDIGTTGNDYSNDVVARAQWF-HNNG---------
Pca_Gewl          QQLRGAVAAYNSGVGN--V-QTIKNMDVGTTGNDYSNDVWARAQALAPSFG---GKADGA
Pyx_gewl          QQLRGAVAAYNSGVGN--V-QTIKNMDVGTTGNDYSNDVWARAQALAPNFG---GKTDGA
Arc_gewl          QQLRGAVAAYNSGVGN--V-QTIKNMDVGTTGNDYSNDVWARAQALAPNFG---GKTDGA
Sa_gewl           QQLRGAVAAYNSGVGN--V-QTLKGMDKGTTGDDYSTDVWARAQELAPHFGGSVGNTGGT
Hya_gewl          QQLRGAVAAYNSGPGN--V-QTIKNMDKGTTGNDYSTDVWARAQELAPHFGGSTGSAGGT
Cg_26612          QHIQGGLAAYNAGIDH--V-DSWSNVDQHTTHHDYSNDVIARAQYLVSHYS---------
Ca_78719          QHIQGGLAAYNAGIDH--V-DSWSNVDQHTTHHDYSNDVIARAQYLVSHYS---------
Se_82739          QQLQGAIAAYNTGPGR--V-SSWNTVDQHTTHGDYSNDVIARAQHLVNNFN---------
Oe_46487          QHIQGGVAAYNFGVDN--V-RSWANLDVGTTGGDYSNDAIARAQWLVTRYK---------
Pc_57781          QKIQGGVAAYNFGVTN--V-RSWEGLDKGTTGNDYSNDVMARAQRL-QALG---------
Pc_57861          QKLQGGVAAYNFGVGN--V-QSWAGLDIGSTGNDYSNDVIARAKYL-KSLG---------
Ls_08512          QKLQGGIAAYNFGVGN--V-QSWTRLDIGSTGNDYSNDVIARAQYL-KSHG---------
Ec_47861          QGLQGGVAAYNKGVSR--V-TSWPGVDTGTTHGDYSNDVIARAQYL-YGLG---------
Ech_17175         QGLQGGVAAYNEGVAN--V-RSWAGLDRGTTHNDYSNDVIARAQYL-YRLG---------
Bg_LYZg2          KQLQGGVAAYNFGVKN--V-KSWAGLDSGSTGNDYSNDVIARAKWL-HLKK---------
Bp_08972          KQLQGGVAAYNFGVKN--V-KSWAGLDSGSTGNDYSNDVIARAKWL-HLKK---------
Bt_15341          RQLQGGVAAYNFGVSN--V-QSWKGLDDGSTGNDYSNDVIARAKWL-YAKG---------
Bt_43005          RQLQGGVAAYNFGPGN--V-RTWEGLDIGSAGNDYSNDVIARAQYL-YSNG---------
Bt_31685          RQLQGGVAAYNFGPGN--V-RTWEGLDIGSAGNDYSNDVIARAQYL-YSNG---------
Bg_LYZg1          RQLQGGVAAYNFGSKN--V-QSWSGLDVGTTNNDYSNDVIARAKYL-YSIG---------
Bp_05359          RQLQGGVAAYNFGSKS--V-KSWSGLDVGSADHDYSNDVIARAKYL-YSIG---------
Bf_05346          RQLQGGVAAYNFGSKN--V-QSWSGLDVGSTNDDYSNDVIARAKYL-YSIG---------
Bg_LYZg3          HQLQGAVAAYNFGTRN--V-QTWAGVDVGTTGGDYSNDVMARAQWL-HNHG---------
```

```
Bp_08986         HQLQGAVAAYNFGTRN--V-QTWAGVDVGTTGGDYSNDVMARAQWL-HNHG---------
Ac_LYZg1         QALQGGVAAYNFNLDN--V-RTWSKLDVGTTHDDYSNDVIARAQYL-HGQG---------
Ec_42267         QCLQGGVAAYNFGVSN--V-QTWGGLDRGSTNDDYSNDVMARAEYL-YNHG---------
Cu_13863         QALQGGVAAYNQGPSN--V-NSWAGVDAATTGRDYSNDVIARAKYL-HSHG---------
Bg_LYZg6         HCLQGAVAGYNCGLSR--V-TSFETADTHTTGHDYSNDVIARAQWL-HQHG---------
Bg_LYZg7         HCLQGAVAGYNCGLSR--V-TSFETADTHTTGHDYSNDVIARAQWL-HQHG---------
Bp_08985         HCLQGAVAGYNCGLSR--V-TTFETADAHTTGHDYSNDVIARAQWL-HQHG---------
Ac_LYZg2         QTLQGGVAAYNFGVSN--V-QSWDRLDIGSTHNDYSGDVIARAQYL-YKHG---------
Pa_37057         QALQGGVAAYNFGVSN--V-QSWGGLDIGSTGNDYSNDVIARAKWL-KANG---------
Pa_34965         QALQGGCAAYNSGVSN--V-QTWGGLDIGTTGNDYSNDVIARAQWL-HHNG---------
Pa_44383         QALQGAVAGYNGGVSR--V-TSWANVDAGTTGHDYSNDVIARAKWL-MANG---------
Bg_LYZg4         QALQGAVAGYNGGVSR--L-ASWSTVDAHTTGHDYSNDVIARAKYL-MAHG---------
Bg_LYZg5         QALQGALAGYNGGVSR--V-TSWADVDAGTTGHDYSNDVVARAKYL-LAHG---------
Se_85121         QQLQGGVAAYNFGVGN--V-QSWGNLDIGSTNNDYSNDVIARAHYLINQHG---------
Pm_29535         QQLQGGIAAYNSGVGN--V-QTWTHLDLGTTGNDYSNDVVARAQRLINHHG---------
My_59246         QQLQGGIAAYNSGVSN--V-QTWAHLDVGTTGNDYSNDVVARAKHLIASHN---------
Mg_gewl          QALQGGVAAYNFGLGN--V-QSWGGLDVGSTHNDYSNDVIARAQWLISHYH---------
Mg_gewl2         QALQGGVAAYNFGLGN--V-QSWGGLDVGSTHNDYSNDVIARAQWLISHYH---------
Lg_LYZg2         YQLKGGVAAYNFGPGN--V-QTIPGMDDKTTGDDYSNDVIARAQRLVKAYG---------
Ls_01764         QQLQGAVSAYNAGTGN--V-ATFSGMDLGTTGNDYSNDVMARAQYLVSHYH---------
Cm_LYZT3a        QQLQGAVSAYNAGTGN--V-RTFDRMDVGTTGNDYSNDVMARAQYLVNHFG---------
Cm_LYZg2         QQLQGAVSAYNAGTGN--V-RTFDRMDEGTTGNDYSNDVMARAQYLVNNFR---------
Ac_LYZg3         QALKGGISAYNAGVGN--V-QTYAGMDLGTTGNDYANDVCARAQRLISHYH---------
Lg_LYZg3         YQLKGGVSAYNAGVGN--V-QTIAGMDAGTTNDDYSNDVIARAQRLVNAHG---------
Hdd_lyzg         QQMQGGVSAYNAGVGN--V-ATWAHLDVGTTGNDYSNDVIARAQHLIKQHG---------
Hr_78614         QQMQGGVSAYNAGVGN--V-ATWSRLDVGTTGNDYSNDVIARAQHLIKQHG---------
Hdi_LysG1        QQMQGGVSAYNAGVGN--V-ATWSHLDVGTTGNDYSNDVIARAQHLINQHG---------
Pm_gewl          QQMQGGVSAYNAGVGN--V-ATVSRMDVGTTGNDYSNDVIARAQRLITAHG---------
Lg_LYZg1         NQLKGGVSAYNAGVGN--V-GTVARMDIGTTGNDYSNDVIARAQRLITSHG---------
Pc_09293         QQMQGGVSAYNAGVGN--V-ATVARMDIGTTGNDYSNDVIARAQRLISAHG---------
Pv_09293         QQMQGGVSAYNAGVGN--V-ATVARMDIGTTGNDYSNDVMARAQRLISAHG---------
                   ::.. ..:. .     :       .      *:  *   *:.

Cc_94995         ------------------------------------------------------------
Cc_98523         ------------------------------------------------------------
Cc_98591         ------------------------------------------------------------
Hs_LYG1          ------------------------------------------------------------
Hs_LYG2          ------------------------------------------------------------
Dg_27517         ------------------------------------------------------------
Xsp_46186        ------------------------------------------------------------
Myx_gewl         ------------------------------------------------------------
Myx_34660        ------------------------------------------------------------
Bf_LYZg6         ------------------------------------------------------------
Bf_LYZg3         ------------------------------------------------------------
La_79286         ------------------------------------------------------------
Bf_LYZg1         ------------------------------------------------------------
Bf_LYZg5         ------------------------------------------------------------
Bf_LYZg4         ------------------------------------------------------------
Bf_LYZg2         ------------------------------------------------------------
Ci_76087         ------------------------------------------------------------
Sc_LYZT3a        ------------------------------------------------------------
Cl_S3624         ------------------------------------------------------------
Cl_10499         ------------------------------------------------------------
Tsp_Lys          ------------------------------------------------------------
Ta_63166         ------------------------------------------------------------
Pca_Gewl         AGPQGPSRPNEPRPSPSAPTLKEGSHGAPVSKLQKQLEKLGFDVGKVDGDFGPKTEAAVK
Pyx_gewl         S---GPSRPNEPRPSPSAPTLKEGSHGAPVTKLQKQLEKLGFDVGKVDGDFGPRTEAAVK
Arc_gewl         S---GPSRPNEPRPSPSAPTLKEGSHGAPVTKLQKQLEKLGFDVGKVDGDFGPRTEAAVK
Sa_gewl          GGTGGTTGPAKP-SGGNSPVLKEGSKGAEVKTLQGRLEKLGFELGKQDGVFGPKTEAAVK
Hya_gewl         GGTGGSTKPSKP-SGGSAPTLREGSKGSEVKSLQSKLEKLGFELGSQDGAFGPKTEAAVK
Cg_26612         ------------------------------------------------------------
Ca_78719         ------------------------------------------------------------
Se_82739         ------------------------------------------------------------
Oe_46487         ------------------------------------------------------------
Pc_57781         ------------------------------------------------------------
Pc_57861         ------------------------------------------------------------
Ls_08512         ------------------------------------------------------------
Ec_47861         ------------------------------------------------------------
Ech_17175        ------------------------------------------------------------
Bg_LYZg2         ------------------------------------------------------------
Bp_08972         ------------------------------------------------------------
```

```
Bt_15341        ------------------------------------------------------------
Bt_43005        ------------------------------------------------------------
Bt_31685        ------------------------------------------------------------
Bg_LYZg1        ------------------------------------------------------------
Bp_05359        ------------------------------------------------------------
Bf_05346        ------------------------------------------------------------
Bg_LYZg3        ------------------------------------------------------------
Bp_08986        ------------------------------------------------------------
Ac_LYZg1        ------------------------------------------------------------
Ec_42267        ------------------------------------------------------------
Cu_13863        ------------------------------------------------------------
Bg_LYZg6        ------------------------------------------------------------
Bg_LYZg7        ------------------------------------------------------------
Bp_08985        ------------------------------------------------------------
Ac_LYZg2        ------------------------------------------------------------
Pa_37057        ------------------------------------------------------------
Pa_34965        ------------------------------------------------------------
Pa_44383        ------------------------------------------------------------
Bg_LYZg4        ------------------------------------------------------------
Bg_LYZg5        ------------------------------------------------------------
Se_85121        ------------------------------------------------------------
Pm_29535        ------------------------------------------------------------
My_59246        ------------------------------------------------------------
Mg_gewl         ------------------------------------------------------------
Mg_gewl2        ------------------------------------------------------------
Lg_LYZg2        ------------------------------------------------------------
Ls_01764        ------------------------------------------------------------
Cm_LYZT3a       ------------------------------------------------------------
Cm_LYZg2        ------------------------------------------------------------
Ac_LYZg3        ------------------------------------------------------------
Lg_LYZg3        ------------------------------------------------------------
Hdd_lyzg        ------------------------------------------------------------
Hr_78614        ------------------------------------------------------------
Hdi_LysG1       ------------------------------------------------------------
Pm_gewl         ------------------------------------------------------------
Lg_LYZg1        ------------------------------------------------------------
Pc_09293        ------------------------------------------------------------
Pv_09293        ------------------------------------------------------------


Cc_94995        ----------------------------------------------IF------------
Cc_98523        ----------------------------------------------F-------------
Cc_98591        ----------------------------------------------IF------------
Hs_LYG1         ----------------------------------------------F-------------
Hs_LYG2         ----------------------------------------------F-------------
Dg_27517        ----------------------------------------------YN------------
Xsp_46186       ----------------------------------------------L-------------
Myx_gewl        ------------GTTTNTDLPGSVTKPGNTQTNTVKPAALPRFNGKYTAAPSLSDVKAGK
Myx_34660       -----------GAPVGGNTTTQPSSTGTNTRPTQTADSFDQGGPKRWTTAPSLAQVQAGG
Bf_LYZg6        ----------------------------------------------F-------------
Bf_LYZg3        ---------------------FGDPKDQRCQGGSNRRRRSSGCPYSWYNPQERPPSGSGS
La_79286        -----------------------FDDPKDQKCQGGSRRRRGTCPYSWFNPNETRPVTSGS
Bf_LYZg1        ----------------------------------------------YN------------
Bf_LYZg5        ----------------------------------------------F-------------
Bf_LYZg4        ----------------------------------------------F-------------
Bf_LYZg2        ----------------------------------------------F-------------
Ci_76087        ----------------------------------------------Y-------------
Sc_LYZT3a       ----------------------------------------------Y-------------
Cl_S3624        ----------------------------------------------Y-------------
Cl_10499        ----------------------------------------------Y-------------
Tsp_Lys         ----------------------------------------------FN------------
Ta_63166        ----------------------------------------------FN------------
Pca_Gewl        RFQAKHHLEADGVVGPKTHAALDKALDHLSKPKPTHSDGFDRGATRWKDAPALADVQSGK
Pyx_gewl        RFQAKHHLEADGVVGPKTHAALDKALDHLSKPKPTHSDGFDNG-SKWKDAPALADVKSGK
Arc_gewl        RFQAKHHLEADGVVGPKTHAALDKALDHLSKPKPTHSDGFDNG-SKWKDAPALADVKSGK
Sa_gewl         RFQSKHNLEVDGITGPKTHQAIEKALSTRAEQAKRQSDSFE-SGSKWKDAPALADVKSGK
Hya_gewl        RFQGKHNLEVDGIAGPKTHAALEKALAARTEQAKLQSDSFDRKATKWKDAPALADVKSGK
Cg_26612        ----------------------------------------------W-------------
Ca_78719        ----------------------------------------------W-------------
Se_82739        ----------------------------------------------W-------------
Oe_46487        ----------------------------------------------W-------------
```

```
Pc_57781        ----------------------------------------------WS------------
Pc_57861        ----------------------------------------------WS------------
Ls_08512        ----------------------------------------------WS------------
Ec_47861        ----------------------------------------------WN------------
Ech_17175       ----------------------------------------------WN------------
Bg_LYZg2        ----------------------------------------------WN------------
Bp_08972        ----------------------------------------------WN------------
Bt_15341        ----------------------------------------------WN------------
Bt_43005        ----------------------------------------------WN------------
Bt_31685        ----------------------------------------------WN------------
Bg_LYZg1        ----------------------------------------------WN------------
Bp_05359        ----------------------------------------------WN------------
Bf_05346        ----------------------------------------------WN------------
Bg_LYZg3        ----------------------------------------------WN------------
Bp_08986        ----------------------------------------------WN------------
Ac_LYZg1        ----------------------------------------------WN------------
Ec_42267        ----------------------------------------------WN------------
Cu_13863        ----------------------------------------------WN------------
Bg_LYZg6        ----------------------------------------------WN------------
Bg_LYZg7        ----------------------------------------------WN------------
Bp_08985        ----------------------------------------------WN------------
Ac_LYZg2        ----------------------------------------------WN------------
Pa_37057        ----------------------------------------------WN------------
Pa_34965        ----------------------------------------------WN------------
Pa_44383        ----------------------------------------------WN------------
Bg_LYZg4        ----------------------------------------------WN------------
Bg_LYZg5        ----------------------------------------------WN------------
Se_85121        ----------------------------------------------W-------------
Pm_29535        ----------------------------------------------WH------------
My_59246        ----------------------------------------------WH------------
Mg_gewl         ----------------------------------------------W-------------
Mg_gewl2        ----------------------------------------------W-------------
Lg_LYZg2        ----------------------------------------------W-------------
Ls_01764        ----------------------------------------------W-------------
Cm_LYZT3a       ----------------------------------------------W-------------
Cm_LYZg2        ----------------------------------------------W-------------
Ac_LYZg3        ----------------------------------------------WDTLPRYEYFQTTE
Lg_LYZg3        ----------------------------------------------W-------------
Hdd_lyzg        ----------------------------------------------W-------------
Hr_78614        ----------------------------------------------W-------------
Hdi_LysG1       ----------------------------------------------W-------------
Pm_gewl         ----------------------------------------------W-------------
Lg_LYZg1        ----------------------------------------------W-------------
Pc_09293        ----------------------------------------------W-------------
Pv_09293        ----------------------------------------------W-------------


Cc_94995        ------------------------------------------------------------
Cc_98523        ------------------------------------------------------------
Cc_98591        ------------------------------------------------------------
Hs_LYG1         ------------------------------------------------------------
Hs_LYG2         ------------------------------------------------------------
Dg_27517        ------------------------------------------------------------
Xsp_46186       ------------------------------------------------------------
Myx_gewl        ADLHIGHQGAAVRELQKKLGVEADGFFGPKTRAAVQKYQLEHGLKPPPGKEGFAGADMLK
Myx_34660       VKLSEGMQGPAVKQIQEMLGIPADGKYGPVTRKAVAEFQKAHDLRPGTAEGHVGPTTL--
Bf_LYZg6        ------------------------------------------------------------
Bf_LYZg3        SGGTHTPGGRHTSGGGGRYVPIVDEFSGPRCSGFRCRQIP--------------------
La_79286        SGGASSRGGSGSTGGSSSRGSSSRHEPIFTPRCVRGRCTQIP------------------
Bf_LYZg1        ------------------------------------------------------------
Bf_LYZg5        ------------------------------------------------------------
Bf_LYZg4        ------------------------------------------------------------
Bf_LYZg2        ------------------------------------------------------------
Ci_76087        ------------------------------------------------------------
Sc_LYZT3a       ------------------------------------------------------------
Cl_S3624        ------------------------------------------------------------
Cl_10499        ------------------------------------------------------------
Tsp_Lys         ------------------------------------------------------------
Ta_63166        ------------------------------------------------------------
Pca_Gewl        AHLQQGMEGGSVKHLQKLLGLETDGKFGPATKKAVADFQREHHLDAGAGRGSVGPKTL--
Pyx_gewl        AHLQQGMEGGSVKHLQKLLGLETDGKFGPATKKAVADFQREHHLDAGAGRGSVGPKTL--
```

```
Arc_gewl        AHLQQGMEGGSVKHLQKLLGLETDGKFGPATKKAVADFQREHHLDAGAGRGSVGPKTL--
Sa_gewl         EHLQQGMEGGSVKHLQKLLGVETDGKFGPNTRKAVAEFQKEHRLDVGDAAGSVGPKTL--
Hya_gewl        EHLQQGMEGGSVKHLQKLLGLETDGKFGPDTKKAVAAFQKDHRLDVGDAAGSVGPKTL--
Cg_26612        ------------------------------------------------------------
Ca_78719        ------------------------------------------------------------
Se_82739        ------------------------------------------------------------
Oe_46487        ------------------------------------------------------------
Pc_57781        ------------------------------------------------------------
Pc_57861        ------------------------------------------------------------
Ls_08512        ------------------------------------------------------------
Ec_47861        ------------------------------------------------------------
Ech_17175       ------------------------------------------------------------
Bg_LYZg2        ------------------------------------------------------------
Bp_08972        ------------------------------------------------------------
Bt_15341        ------------------------------------------------------------
Bt_43005        ------------------------------------------------------------
Bt_31685        ------------------------------------------------------------
Bg_LYZg1        ------------------------------------------------------------
Bp_05359        ------------------------------------------------------------
Bf_05346        ------------------------------------------------------------
Bg_LYZg3        ------------------------------------------------------------
Bp_08986        ------------------------------------------------------------
Ac_LYZg1        ------------------------------------------------------------
Ec_42267        ------------------------------------------------------------
Cu_13863        ------------------------------------------------------------
Bg_LYZg6        ------------------------------------------------------------
Bg_LYZg7        ------------------------------------------------------------
Bp_08985        ------------------------------------------------------------
Ac_LYZg2        ------------------------------------------------------------
Pa_37057        ------------------------------------------------------------
Pa_34965        ------------------------------------------------------------
Pa_44383        ------------------------------------------------------------
Bg_LYZg4        ------------------------------------------------------------
Bg_LYZg5        ------------------------------------------------------------
Se_85121        ------------------------------------------------------------
Pm_29535        ------------------------------------------------------------
My_59246        ------------------------------------------------------------
Mg_gewl         ------------------------------------------------------------
Mg_gewl2        ------------------------------------------------------------
Lg_LYZg2        ------------------------------------------------------------
Ls_01764        ------------------------------------------------------------
Cm_LYZT3a       ------------------------------------------------------------
Cm_LYZg2        ------------------------------------------------------------
Ac_LYZg3        SDDRPNPAVMGTPFYVLNLAVLPPRPDPIPQWSPRTISTVFIYQPFPSSRSGGYGNGG--
Lg_LYZg3        ------------------------------------------------------------
Hdd_lyzg        ------------------------------------------------------------
Hr_78614        ------------------------------------------------------------
Hdi_LysG1       ------------------------------------------------------------
Pm_gewl         ------------------------------------------------------------
Lg_LYZg1        ------------------------------------------------------------
Pc_09293        ------------------------------------------------------------
Pv_09293        ------------------------------------------------------------


Cc_94995        ------------------------------------------------------------
Cc_98523        ------------------------------------------------------------
Cc_98591        ------------------------------------------------------------
Hs_LYG1         ------------------------------------------------------------
Hs_LYG2         ------------------------------------------------------------
Dg_27517        ------------------------------------------------------------
Xsp_46186       ------------------------------------------------------------
Myx_gewl        KLGITNKPATKPTGDSFETTNTKPKPGTTQTQTDNTTNKPGAVLGNGVTIDTNNPTLKKL
Myx_34660       -RTLQGNRGTNTGNTTGGTNTGNTTNGTNTGNSTTATNNNGAVLGNGVRINTNDPTLKKL
Bf_LYZg6        ------------------------------------------------------------
Bf_LYZg3        ------------------------------------------------------------
La_79286        ------------------------------------------------------------
Bf_LYZg1        ------------------------------------------------------------
Bf_LYZg5        ------------------------------------------------------------
Bf_LYZg4        ------------------------------------------------------------
Bf_LYZg2        ------------------------------------------------------------
Ci_76087        ------------------------------------------------------------
```

```
Sc_LYZT3a       ------------------------------------------------------------
Cl_S3624        ------------------------------------------------------------
Cl_10499        ------------------------------------------------------------
Tsp_Lys         ------------------------------------------------------------
Ta_63166        ------------------------------------------------------------
Pca_Gewl        -AAMEKAATSDGPGNATPAMRKLAK-----------------------------------
Pyx_gewl        -AAMEKAARSDGPGDATPAMRKLAK-----------------------------------
Arc_gewl        -AAMEKAARSDGPGDATPAMRKLAK-----------------------------------
Sa_gewl         -AAMEKAARSQGPGNID-------------------------------------------
Hya_gewl        -AAMEKAAKSSGGGAID-------------------------------------------
Cg_26612        ------------------------------------------------------------
Ca_78719        ------------------------------------------------------------
Se_82739        ------------------------------------------------------------
Oe_46487        ------------------------------------------------------------
Pc_57781        ------------------------------------------------------------
Pc_57861        ------------------------------------------------------------
Ls_08512        ------------------------------------------------------------
Ec_47861        ------------------------------------------------------------
Ech_17175       ------------------------------------------------------------
Bg_LYZg2        ------------------------------------------------------------
Bp_08972        ------------------------------------------------------------
Bt_15341        ------------------------------------------------------------
Bt_43005        ------------------------------------------------------------
Bt_31685        ------------------------------------------------------------
Bg_LYZg1        ------------------------------------------------------------
Bp_05359        ------------------------------------------------------------
Bf_05346        ------------------------------------------------------------
Bg_LYZg3        ------------------------------------------------------------
Bp_08986        ------------------------------------------------------------
Ac_LYZg1        ------------------------------------------------------------
Ec_42267        ------------------------------------------------------------
Cu_13863        ------------------------------------------------------------
Bg_LYZg6        ------------------------------------------------------------
Bg_LYZg7        ------------------------------------------------------------
Bp_08985        ------------------------------------------------------------
Ac_LYZg2        ------------------------------------------------------------
Pa_37057        ------------------------------------------------------------
Pa_34965        ------------------------------------------------------------
Pa_44383        ------------------------------------------------------------
Bg_LYZg4        ------------------------------------------------------------
Bg_LYZg5        ------------------------------------------------------------
Se_85121        ------------------------------------------------------------
Pm_29535        ------------------------------------------------------------
My_59246        ------------------------------------------------------------
Mg_gewl         ------------------------------------------------------------
Mg_gewl2        ------------------------------------------------------------
Lg_LYZg2        ------------------------------------------------------------
Ls_01764        ------------------------------------------------------------
Cm_LYZT3a       ------------------------------------------------------------
Cm_LYZg2        ------------------------------------------------------------
Ac_LYZg3        -SSYGILQCNSKTTSLDCDKYAWDS-----------------------------------
Lg_LYZg3        ------------------------------------------------------------
Hdd_lyzg        ------------------------------------------------------------
Hr_78614        ------------------------------------------------------------
Hdi_LysG1       ------------------------------------------------------------
Pm_gewl         ------------------------------------------------------------
Lg_LYZg1        ------------------------------------------------------------
Pc_09293        ------------------------------------------------------------
Pv_09293        ------------------------------------------------------------


Cc_94995        ------------------------------------------------------------
Cc_98523        ------------------------------------------------------------
Cc_98591        ------------------------------------------------------------
Hs_LYG1         ------------------------------------------------------------
Hs_LYG2         ------------------------------------------------------------
Dg_27517        ------------------------------------------------------------
Xsp_46186       ------------------------------------------------------------
Myx_gewl        ATSRLDNGPTGYCVQTTLNNMRRLGIPNTPSATGNDPNNPRGGMAQMLRNGWESIPFPGS
Myx_34660       ATSHLNNGATGYCVRTTLDNMTRLGIPNTPAATGNDPNNPRGGMAQMLRKGWDSIPFPGS
Bf_LYZg6        ------------------------------------------------------------
```

```
Bf_LYZg3        ------------------------------------------------------------
La_79286        ------------------------------------------------------------
Bf_LYZg1        ------------------------------------------------------------
Bf_LYZg5        ------------------------------------------------------------
Bf_LYZg4        ------------------------------------------------------------
Bf_LYZg2        ------------------------------------------------------------
Ci_76087        ------------------------------------------------------------
Sc_LYZT3a       ------------------------------------------------------------
Cl_S3624        ------------------------------------------------------------
Cl_10499        ------------------------------------------------------------
Tsp_Lys         ------------------------------------------------------------
Ta_63166        ------------------------------------------------------------
Pca_Gewl        -------------AGHAAAMSIGGYNSQGLCATGVSKAIQNAFGFKV------WGNGNQI
Pyx_gewl        -------------AGHAAAMSIGGYNSQGLCATGVSKAIQNAFGFKV------WGNGNQI
Arc_gewl        -------------AGHAAAMSIGGYNSQGLCATGVSKAIQNAFGFKV------WGNGNQI
Sa_gewl         -----------------AGRGWGG--SEGVADA--AKAIARDMGIPVTSQKRNLADTKRV
Hya_gewl        -----------------DGRGWGG--SEGVADA--AKAIAREMGIPVTSQKRNLADTKRV
Cg_26612        ------------------------------------------------------------
Ca_78719        ------------------------------------------------------------
Se_82739        ------------------------------------------------------------
Oe_46487        ------------------------------------------------------------
Pc_57781        ------------------------------------------------------------
Pc_57861        ------------------------------------------------------------
Ls_08512        ------------------------------------------------------------
Ec_47861        ------------------------------------------------------------
Ech_17175       ------------------------------------------------------------
Bg_LYZg2        ------------------------------------------------------------
Bp_08972        ------------------------------------------------------------
Bt_15341        ------------------------------------------------------------
Bt_43005        ------------------------------------------------------------
Bt_31685        ------------------------------------------------------------
Bg_LYZg1        ------------------------------------------------------------
Bp_05359        ------------------------------------------------------------
Bf_05346        ------------------------------------------------------------
Bg_LYZg3        ------------------------------------------------------------
Bp_08986        ------------------------------------------------------------
Ac_LYZg1        ------------------------------------------------------------
Ec_42267        ------------------------------------------------------------
Cu_13863        ------------------------------------------------------------
Bg_LYZg6        ------------------------------------------------------------
Bg_LYZg7        ------------------------------------------------------------
Bp_08985        ------------------------------------------------------------
Ac_LYZg2        ------------------------------------------------------------
Pa_37057        ------------------------------------------------------------
Pa_34965        ------------------------------------------------------------
Pa_44383        ------------------------------------------------------------
Bg_LYZg4        ------------------------------------------------------------
Bg_LYZg5        ------------------------------------------------------------
Se_85121        ------------------------------------------------------------
Pm_29535        ------------------------------------------------------------
My_59246        ------------------------------------------------------------
Mg_gewl         ------------------------------------------------------------
Mg_gewl2        ------------------------------------------------------------
Lg_LYZg2        ------------------------------------------------------------
Ls_01764        ------------------------------------------------------------
Cm_LYZT3a       ------------------------------------------------------------
Cm_LYZg2        ------------------------------------------------------------
Ac_LYZg3        -------------CQHINFMVHSFLIPRIKAVRQAHPSWTQDQTLQAGISVYNTGVTNVQ
Lg_LYZg3        ------------------------------------------------------------
Hdd_lyzg        ------------------------------------------------------------
Hr_78614        ------------------------------------------------------------
Hdi_LysG1       ------------------------------------------------------------
Pm_gewl         ------------------------------------------------------------
Lg_LYZg1        ------------------------------------------------------------
Pc_09293        ------------------------------------------------------------
Pv_09293        ------------------------------------------------------------


Cc_94995        ------------------------------------------------------------
Cc_98523        ------------------------------------------------------------
Cc_98591        ------------------------------------------------------------
```

```
Hs_LYG1         ------------------------------------------------------------
Hs_LYG2         ------------------------------------------------------------
Dg_27517        ------------------------------------------------------------
Xsp_46186       ------------------------------------------------------------
Myx_gewl        KQKAIKSPYGNATANVVTADQYRQLVKEGKVPDGAIIFQTRHGWDYSGGSKGNDMGVVRN
Myx_34660       RQQTIKSPYGNATANVVTADQYKKLVAEGKVPDGAIIFQSRHGWDYSGGSKGNDMGVVRN
Bf_LYZg6        ------------------------------------------------------------
Bf_LYZg3        ------------------------------------------------------------
La_79286        ------------------------------------------------------------
Bf_LYZg1        ------------------------------------------------------------
Bf_LYZg5        ------------------------------------------------------------
Bf_LYZg4        ------------------------------------------------------------
Bf_LYZg2        ------------------------------------------------------------
Ci_76087        ------------------------------------------------------------
Sc_LYZT3a       ------------------------------------------------------------
Cl_S3624        ------------------------------------------------------------
Cl_10499        ------------------------------------------------------------
Tsp_Lys         ------------------------------------------------------------
Ta_63166        ------------------------------------------------------------
Pca_Gewl        DNNLPRDKFKQVHMSLAEALKTPGLVLTWEKTSTAAGSKYGHTAITTGDGHSSVSDFIER
Pyx_gewl        DNNLPRDKFKQVHMSLAEALKTPGLVLTWEKTSTTAGQKYGHTAITTGDGHSSVSDFIER
Arc_gewl        DNNLPRDKFKQVHMSLAEALKTPGLVLTWEKTSTTAGQKYGHTAITTGDGHSSVSDFIER
Sa_gewl         GSTTGSDHYTGNKNAFATDFGVSG--QRGDQLARAIAKKYGIPASNIGT--------YNR
Hya_gewl        GSTTGSDHYTGNKNAFATDFGVAG--ARGDQLARAIAKKYGIPASNIGT--------YNR
Cg_26612        ------------------------------------------------------------
Ca_78719        ------------------------------------------------------------
Se_82739        ------------------------------------------------------------
Oe_46487        ------------------------------------------------------------
Pc_57781        ------------------------------------------------------------
Pc_57861        ------------------------------------------------------------
Ls_08512        ------------------------------------------------------------
Ec_47861        ------------------------------------------------------------
Ech_17175       ------------------------------------------------------------
Bg_LYZg2        ------------------------------------------------------------
Bp_08972        ------------------------------------------------------------
Bt_15341        ------------------------------------------------------------
Bt_43005        ------------------------------------------------------------
Bt_31685        ------------------------------------------------------------
Bg_LYZg1        ------------------------------------------------------------
Bp_05359        ------------------------------------------------------------
Bf_05346        ------------------------------------------------------------
Bg_LYZg3        ------------------------------------------------------------
Bp_08986        ------------------------------------------------------------
Ac_LYZg1        ------------------------------------------------------------
Ec_42267        ------------------------------------------------------------
Cu_13863        ------------------------------------------------------------
Bg_LYZg6        ------------------------------------------------------------
Bg_LYZg7        ------------------------------------------------------------
Bp_08985        ------------------------------------------------------------
Ac_LYZg2        ------------------------------------------------------------
Pa_37057        ------------------------------------------------------------
Pa_34965        ------------------------------------------------------------
Pa_44383        ------------------------------------------------------------
Bg_LYZg4        ------------------------------------------------------------
Bg_LYZg5        ------------------------------------------------------------
Se_85121        ------------------------------------------------------------
Pm_29535        ------------------------------------------------------------
My_59246        ------------------------------------------------------------
Mg_gewl         ------------------------------------------------------------
Mg_gewl2        ------------------------------------------------------------
Lg_LYZg2        ------------------------------------------------------------
Ls_01764        ------------------------------------------------------------
Cm_LYZT3a       ------------------------------------------------------------
Cm_LYZg2        ------------------------------------------------------------
Ac_LYZg3        TVAGVDSSTQGRDFSNDVCARAQRLINHHKWE----------------------------
Lg_LYZg3        ------------------------------------------------------------
Hdd_lyzg        ------------------------------------------------------------
Hr_78614        ------------------------------------------------------------
Hdi_LysG1       ------------------------------------------------------------
Pm_gewl         ------------------------------------------------------------
Lg_LYZg1        ------------------------------------------------------------
```

```
Pc_09293        ------------------------------------------------------------
Pv_09293        ------------------------------------------------------------


Cc_94995        ----------------------------------
Cc_98523        ----------------------------------
Cc_98591        ----------------------------------
Hs_LYG1         ----------------------------------
Hs_LYG2         ----------------------------------
Dg_27517        ----------------------------------
Xsp_46186       ----------------------------------
Myx_gewl        NGKTTHNYADMSSIIYSDCKEVAILVPKDAIKRD
Myx_34660       GGKTTHNYKDMPSIIYSDCKEVVILVPKGAIQRD
Bf_LYZg6        ----------------------------------
Bf_LYZg3        ----------------------------------
La_79286        ----------------------------------
Bf_LYZg1        ----------------------------------
Bf_LYZg5        ----------------------------------
Bf_LYZg4        ----------------------------------
Bf_LYZg2        ----------------------------------
Ci_76087        ----------------------------------
Sc_LYZT3a       ----------------------------------
Cl_S3624        ----------------------------------
Cl_10499        ----------------------------------
Tsp_Lys         ----------------------------------
Ta_63166        ----------------------------------
Pca_Gewl        NTLGAGGR----------------TGLKIFRPTI
Pyx_gewl        NTLNASGR----------------TGLKIFRPTI
Arc_gewl        NTLNASGR----------------TGLKIFRPTI
Sa_gewl         HTINVDGQKYSLQLLWKVKGHFDHVHLGIQRAN-
Hya_gewl        HTINVDGQKYSLQLLWKVKGHFDHVHLGIQKH--
Cg_26612        ----------------------------------
Ca_78719        ----------------------------------
Se_82739        ----------------------------------
Oe_46487        ----------------------------------
Pc_57781        ----------------------------------
Pc_57861        ----------------------------------
Ls_08512        ----------------------------------
Ec_47861        ----------------------------------
Ech_17175       ----------------------------------
Bg_LYZg2        ----------------------------------
Bp_08972        ----------------------------------
Bt_15341        ----------------------------------
Bt_43005        ----------------------------------
Bt_31685        ----------------------------------
Bg_LYZg1        ----------------------------------
Bp_05359        ----------------------------------
Bf_05346        ----------------------------------
Bg_LYZg3        ----------------------------------
Bp_08986        ----------------------------------
Ac_LYZg1        ----------------------------------
Ec_42267        ----------------------------------
Cu_13863        ----------------------------------
Bg_LYZg6        ----------------------------------
Bg_LYZg7        ----------------------------------
Bp_08985        ----------------------------------
Ac_LYZg2        ----------------------------------
Pa_37057        ----------------------------------
Pa_34965        ----------------------------------
Pa_44383        ----------------------------------
Bg_LYZg4        ----------------------------------
Bg_LYZg5        ----------------------------------
Se_85121        ----------------------------------
Pm_29535        ----------------------------------
My_59246        ----------------------------------
Mg_gewl         ----------------------------------
Mg_gewl2        ----------------------------------
Lg_LYZg2        ----------------------------------
Ls_01764        ----------------------------------
Cm_LYZT3a       ----------------------------------
Cm_LYZg2        ----------------------------------
```

```
Ac_LYZg3        ----------------------------------
Lg_LYZg3        ----------------------------------
Hdd_lyzg        ----------------------------------
Hr_78614        ----------------------------------
Hdi_LysG1       ----------------------------------
Pm_gewl         ----------------------------------
Lg_LYZg1        ----------------------------------
Pc_09293        ----------------------------------
Pv_09293        ----------------------------------
```
